# The radical scavenging activity of monocaffeoylquinic acids: the role of neighboring hydroxyl groups and pH levels†

**DOI:** 10.1039/d3ra08460d

**Published:** 2024-01-30

**Authors:** Quan V. Vo, Duong Thi Thuy Hoa, Nguyen Thi Hoa, Manh Duc Tran, Adam Mechler

**Affiliations:** a The University of Danang – University of Technology and Education Danang 550000 Vietnam vvquan@ute.udn.vn; b The University of Danang – University of Sciences and Education Danang 550000 Vietnam; c Department of Biochemistry and Chemistry, La Trobe University Victoria 3086 Australia

## Abstract

Caffeoylquinic acids (CQAs) are well-known antioxidants. However, a key aspect of their radical scavenging activity – the mechanism of action – has not been addressed in detail thus far. Here we report on a computational study of the mechanism of activity of CQAs in scavenging hydroperoxyl radicals. In water at physiological pH, the CQAs demonstrated ≈ 10^4^ times higher HOO˙ antiradical activity than in lipid medium (*k*_(lipid)_ ≈ 10^4^ M^−1^ s^−1^). The activity in the aqueous solution was determined by the hydrogen transfer mechanism of the adjacent hydroxyl group (O6′–H) of the dianion states (*Γ* = 93.2–95.2%), while the single electron transfer reaction of these species contributed 4.8–6.8% to the total rate constants. The kinetics estimated by the calculations are consistent with experimental findings in water (pH = 7.5), yielding a *k*_calculated_/*k*_experimental_ = 2.4, reinforcing the reliability and precision of the computational method and demonstrating its utility for evaluating radical reactions *in silico*. The results also revealed the pH dependence of the HOO˙ scavenging activity of the CQAs; activity was comparable for all compounds below pH 3, however at higher pH values 5CQA reacted with the HOO˙ with lower activity than 3CQA or 4CQA. It was also found that CQAs are less active than Trolox below pH 4.7, however over pH 5.0 they showed higher activity than the reference. The CQAs had the best HOO˙ antiradical activity at pH values between 5.0 and 8.6. Therefore, in the physiological environment, the hydroperoxyl antiradical capacity of CQAs exhibits similarity to renowned natural antioxidants including resveratrol, ascorbic acid, and Trolox.

## Abstract 2

Through the hydrogen transfer reaction of adjacent hydroxyl groups, CQAs demonstrate excellent antioxidant activity in aqueous physiological environments.

## Graphical abstract

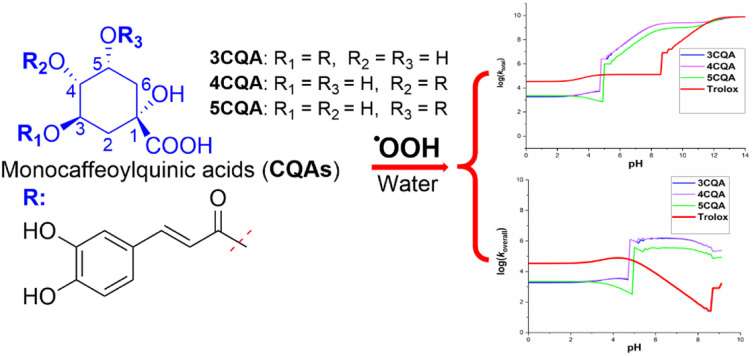

## 1. Introduction

**Fig. 1:**
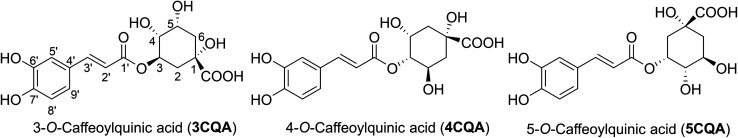
Monocaffeoylquinic acids (CQAs).

Caffeoylquinic acids (CQAs) are a class of bioactive metabolites that are synthesized through the phenylpropanoid biosynthesis pathway.^1^ These compounds are esters formed by the conjugation of caffeic acid and quinic acid. CQAs are frequently found in a diverse array of food sources, encompassing fruits, coffee, vegetables, spices, and an extensive variety of plant species.^2,3^CQAs have a diverse array of potential therapeutic uses in humans. It has been reported in a range of studies that these compounds possess antibacterial, anticancer, antiviral, anti-Alzheimer's, neuroprotective, and antioxidant properties.^1,4–14^ The most common CQA in the plant kingdom is 5-*O*-caffeoylquinic acid (5CQA), also known as chlorogenic acid. This compound is usually found in combination with 3-*O*-caffeoylquinic acid (3CQA), also known as neochlorogenic acid, and 4-*O*-caffeoylquinic acid (4CQA), also known as cryptochlorogenic acid (Fig. 1).^15^

Chlorogenic acids have the ability to scavenge free radicals from a variety of sources, including 2,21-azino-bis(3-ethylbenzothiazoline-6-sulphonic acid) (ABTS) radicals, 1,1-diphenyl-2-picrylhydrazyl (DPPH) radicals, superoxide anions (O2), hydroxyl radicals (OH) and peroxynitrite (ONOO).^13,16,17^ Kinetics of 5CQA were determined experimentally: it reacts with the superoxide, peroxynitrite, and peroxyl radical with second-order kinetics and rate constants of 3.34 × 10^9^, 9.60 × 10^5^, 1.6 × 10^5^ and 1.28 × 10^5^ M^−1^ s^−1^, respectively,^17^ whereas the 5CQA reacted with HO˙ radicals with rate constant of 10^9^–10^10^ M^−1^ s^−1^.^18,19^ Computational approaches were also used to evaluate the antioxidant activity of CQAs,^18–23^ however the mechanism and kinetics of the HOO˙ radical scavenging activity, especially in physiological environments and at different pH values have not been thoroughly investigated. In particular, the effect of different pH levels on the kinetics and mechanism of the radical scavenging activity of phenolic acids is well known,^24–26^ and thus a more comprehensive examination is desirable. Furthermore, the examination of radical reactions involving HO˙ and HOO˙ is significant not only in the advanced oxidation processes,^27–29^ but also in the radical scavenging activity of antioxidants.^30–33^ Accordingly, in this study, thermodynamic and kinetic calculations were used to examine the hydroperoxyl radical scavenging activity of the CQAs in physiological conditions and at varied pH levels.

## 2. Computational details

The thermochemical characteristics (bond dissociation energies (BDEs), ionization energies (IEs), and proton affinities (PAs)) of the compound were investigated at the M06-2X/6-311++G(d,p) level of theory. Additionally, the kinetic parameters of the compound, including their activation energies (Δ*G*^≠^) in kcal mol^−1^, tunneling corrections (*κ*), and rate constants (*k*), were computed. The activities of the compounds were modelled in the gas phase, the physiological environment (the lipid medium consisted of pentyl ethanoate). In comparison to other intricate procedures, such as G3(MP2)-RAD, and empirical data, it has been demonstrated that the M06-2X/6-311++G(d,p) method has appropriate accuracy in the computation of thermodynamic properties, with an acceptable margin of error^34–38^ and overall low error rates (*k*_calc_/*k*_exp_ ratio = 0.3–2.9).^39–42^ The kinetic calculations were conducted following the established technique for the quantum mechanics-based assay designed to evaluate the overall free radical scavenging activity (QM-ORSA) with the solvation model based on the density (SMD) method for pentyl ethanoate and water solvents. The aforementioned test has been widely employed in order to assess the antiradical properties of antioxidants.^34,35,39,40,43^

The rate constant (*k*) was determined through the application of the usual transition state theory (TST) under the conditions of a 1 M standard state,^44–48^ and the details are shown in Table S1, ESI.†1
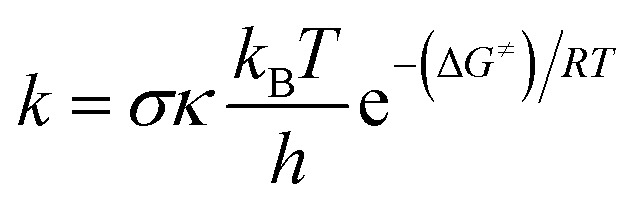
Here Δ*G*^≠^ represents the Gibbs free energy of activation, *h* denotes the Planck constant, *k*_B_ represents the Boltzmann constant, *s* is the reaction symmetry number,^49,50^ and *κ* signifies the tunneling corrections that were determined by the utilization of the Eckart barrier calculation method,.^51^

The computations were performed utilizing the Gaussian 16 suite of programs^52^ and the Eyringpy code, depending on the particular circumstance.^53,54^ Atom-in-molecule (AIM) analysis^55^ was performed by using the AIM2000 software.^56^

## 3. Results and discussion

### 3.1. The thermodynamic study

Based on the core structure of CQA, the hexagon rings, HO, and COOH groups can undergo rotation to yield a variety of conformers. The most likely conformer to participate in a radical scavenging reaction is the most stable one, and thus electron energy levels of all possible conformers of each CQA were evaluated in the first stage.^57^ Subsequently, the five conformers with the lowest electronic energy were subjected to free energy analysis using the M06-2X/6-311++G (d,p) level of theory. Details are shown in the ESI (Fig. S2).† It was found that the Δ*G*° value of 3CQA (*i.e.* the structure as drawn in Fig. S2, ESI†) was determined to be the lowest among all the 3CQA conformers (3CQA-1–4) by 2.8–5.1 kcal mol^−1^. Similarly, the lowest energy 4CQA and 5CQA conformers are drawn in Fig. S2, ESI.† The estimation of conformer relative populations using the Maxwell–Boltzmann distribution^58,59^ revealed that the conformers 3CQA, 4CQA, and 5CQA dominate the populations (>95%) under standard conditions; consequently, these conformers were used in subsequent investigations.

**Fig. 2:**
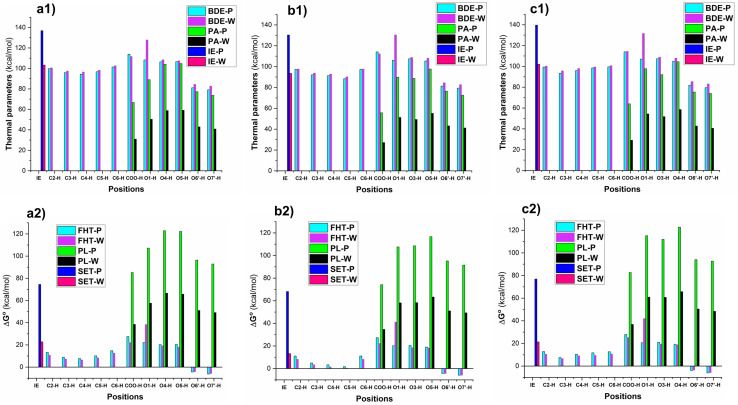
The computed BDE, PA, IE (kcal mol^−1^) of CQAs and Δ*G*° (kcal mol^−1^) of the HOO˙ + CQAs reactions following the SET, PL, and FHT mechanisms (a: 3CQA; b: 4CQA; c: 5CQA).

In order to evaluate the likelihood of reacting with free radicals of all possible X–H (X = C, O) bonds including C–H (C2, C3, C4, C5 and C6), and O–H (COOH, O1, O3, O5, O6′ and O7′, Fig. 1) key thermochemical characteristics: bond dissociation energies (BDEs), proton affinities (PAs), and ionization energies (IEs) that provide a first approximation for the probability of reactions following either of the three respective mechanisms, *i.e.* FHT (formal hydrogen transfer), SET (single electron transfer) and PL (proton loss),^41,60,61^ were first computed in physiological environments (and water (W) and pentyl ethanoate (P)). The results are shown in Fig. 2.

The lowest BDE values were observed at the O6′(7′)–H bonds in all of the studied acids with BDE(O6′(7′)–H) = 79.1–81.9 kcal mol^−1^ for the lipid medium and BDE(O6′(7′)–H) = 82.7–85.4 kcal mol^−1^ for the aqueous solution (Fig. 2a1–c1). The active site can be attributed in all cases to the formation of intramolecular hydrogen bonds between the hydrogen atoms of the adjacent hydroxyl groups and the O6′(O7′) radicals.^60,62^ While the values for other O–H bonds ranged from 104.9 to 131.6 kcal mol^−1^, the BDE(C–H) values were between 88.3 and 102.6 kcal mol^−1^. Surprisingly the H-abstraction of the COO–H bond was less likely with the BDE(COO–H) = 112.3–114.4 kcal mol^−1^ (to emphasize, this refers to hydrogen abstraction; proton dissociation is much more likely, see below). The BDE values in the water were slightly higher than those in the pentyl ethanoate solvent. As expected the PA and IE values in the polar medium were lower than those of the nonpolar environment. The deprotonation was in the order of COO–H > O7′–H > O6′–H in all of the studied compounds, whereas the IE values varied from 93.5 to 139.6 kcal mol^−1^.

The evaluation in the Gibbs free energies (Δ*G*°, Fig. 2a2–c2) of the HOO˙ + CQAs reactions following either of the three pathways revealed that the HOO˙ radical trapping activity of CQAs is only spontaneous *via* the hydrogen transfer of the O6′(7′)-H bonds (Δ*G*° = −3.6 to −6.2 kcal mol^−1^), whereas the other FHT reactions cannot happen in the studied media due to the positive Δ*G*° values. The SET mechanism is not spontaneous either in any of the studied environments, thus this reaction of the neutral states of CQAs can be safely ignored in the kinetic study. It is important to notice that the PL reactions are not spontaneous either in any of the studied environments; however, the PA values were substantially lower than the corresponding BDE values, thus the deprotonation of CQAs should be considered in the aqueous solution. Previous studies indicated that the addition reaction into the α,β-unsaturated bond had no contributions to the ROO˙ radical (*i.e.*, HOO˙ and CH_3_OO˙) scavenging activity, particularly in the physiological environments,^19,24,63^ and RAF reaction is not supported for the π system of aromatic rings.^64,65^ Thus, this reaction was omitted in our study. Hence, in the lipid medium, the H-abstraction of the O6′(7′)–H bonds should be used to compute rate constants, whereas, in the aqueous solution, proton dissociation should be assessed before the kinetic investigation.

### 3.2. The kinetics of antioxidant activity

#### 3.2.1. The deprotonation of CQA

**Fig. 3:**
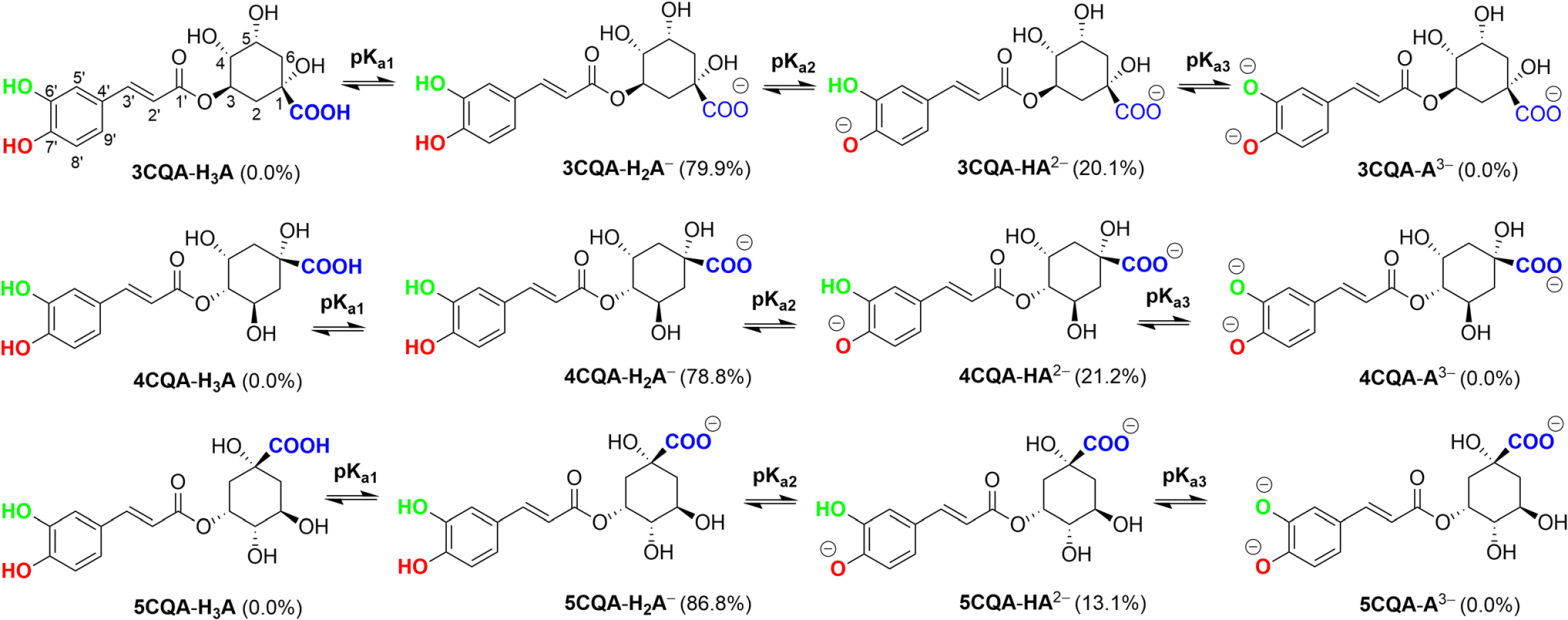
The deprotonation of CQAs in water under pH = 7.40.

The dissociated form of acidic species frequently overshadows the antiradical activity of the neutral species in aqueous environments.^37,41^ Thus the protonation states of CQAs in water at the physiological pH were analyzed. The structure of CQAs permits protonation at the COOH (p*K*_a1_), O7′–H (p*K*_a2_), and O6′–H (p*K*_a3_) bonds (Fig. 2); the p*K*_a1_ values of CQAs were obtained from a previous study,^66^ while the p*K*_a2_ and p*K*_a3_ values were computed according to the previous study.^30^ The data are displayed in Fig. 3 and Table 1.

**Table 1:** Calculated p*K*_a_ and *f*

Comp.	Group	p*K*_a_		*f* (pH = 7.40)^c^	
3CQA	COOH	1	3.95^a^	H_3_A	0.000
	O6′–H	2	8.00^b^	H_2_A^−^	0.799
	O7′–H	3	12.59^b^	HA^2−^	0.201
				A^3−^	0.000
4CQA	COOH	1	4.14^a^	H_3_A	0.000
	O6′–H	2	7.97^b^	H_2_A^−^	0.788
	O7′–H	3	12.52^b^	HA^2−^	0.212
				A^3−^	0.000
5CQA	COOH	1	3.95^a^	H_3_A	0.000
	O6′–H	2	8.22^b^	H_2_A^−^	0.868
	O7′–H	3	12.27^b^	HA^2−^	0.131
				A^3−^	0.000

a Ref. 66.

b Calculated in this work.

c *f* Molar fraction.

The p*K*_a1_ values for 3CQA and 5CQA are 3.95, while those for 4CQA are 4.14 (Table 1). The range of p*K*_a2_ values is 7.97 to 8.22, while the range of p*K*_a3_ values is 12.27 to 12.59. The calculated p*K*_a_ values of 5CQA (p*K*_a2_ = 8.22 and p*K*_a3_ = 12.27) closely align with the experimental results (p*K*_a2_ = 8.21 and p*K*_a3_ = 12.5),^67^ providing evidence for the accuracy and validity of the computational approach. The mole fractions *f*(H_2_A^−^) and *f*(HA^2−^) range between 0.788 and 0.868 and between 0.131 and 0.212, respectively, while the H_3_A and A^3−^ phases are not present in water at pH = 7.40. Therefore, the CQAs exist in both anionic and dianionic states in water with a pH of 7.4. These two states were examined in the subsequent investigation.

#### 3.2.2. The kinetics of the reaction of CQAs with HOO˙ radical in the physiological environments

**Fig. 4:**
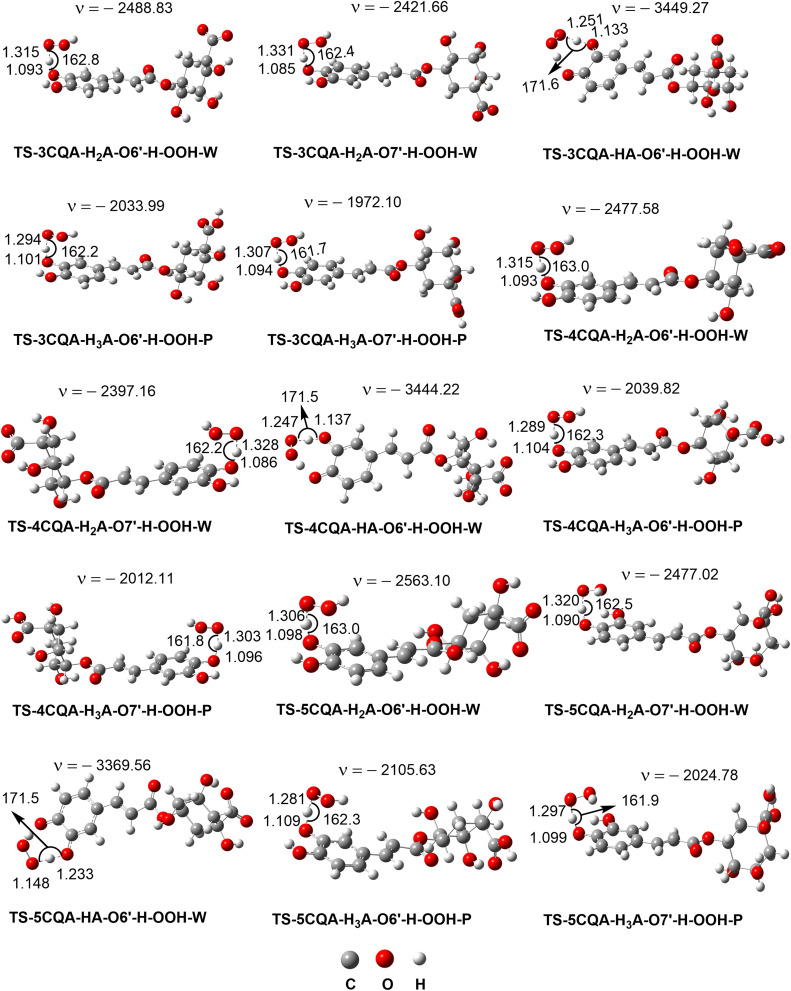
The FHT TS structures of the HOO˙ + CQAs reactions (W: water; P: pentyl ethanoate).

The kinetics of the reactions between CQAs and HOO˙ in the aqueous solution were investigated for all states, using the methodology employed in earlier research on phenolic compounds. The competitive FHT reaction was utilized to evaluate the kinetics for neutral states, while the SET reaction was employed for anion states.^25,31,35^ Using eqn (2) and (3), the total rate constants of the states (*k*_total_) were determined, whereas eqn (4) was used to derive the rate constant containing the molar fraction (*k*_f_). Fig. 4 depicts the optimized transition structures (TS), data are in Table 2.

**Table 2:** Computed Δ*G*^≠^ (kcal mol^−1^) *Γ* (%), *k*_app_, *k*_f_, and *k*_overall_ (M^−1^ s^−1^) of the CQAs + HOO˙ reactions in the studied media

Comp.	Mechanisms		Pentyl ethanoate			Water					
			Δ*G*^≠^	*k*_app_	*Γ*	States	Δ*G*^≠^	*k*_app_	*F*	*k*_f_	*Γ*
3CQA	SET					H_2_A^−^	34.3	15.9	0.799	3.84 × 10^−13^	0.0
						HA^2−^	6.4	1.20 × 10^8^	0.201	2.41 × 10^7^	4.8
	FHT	O6′–H	15.3	6.30 × 10^3^	57.8	H_2_A^−^	15.9	4.80 × 10^3^	0.799	3.84 × 10^3^	0.0
		O7′–H	15.2	4.60 × 10^3^	42.2		16.6	1.00 × 10^3^	0.799	7.99 × 10^2^	0.0
		O6′–H (HA^2−^)				HA^2−^	1.0	2.40 × 10^9^	0.201	4.82 × 10^8^	95.2
	*k*_total_			**1.09 × 10** ^**4**^						**5.07 × 10** ^**8**^	
4CQA	SET					H_2_A^−^	32.3	17.2	0.788	1.10 × 10^−8^	0.0
						HA^2−^	6.4	1.30 × 10^8^	0.212	2.76 × 10^7^	5.2
	FHT	O6′–H	14.9	8.00 × 10^2^	4.8	H_2_A^−^	16.2	2.10 × 10^3^	0.788	1.65 × 10^3^	0.0
		O7′–H	14.6	1.60 × 10^4^	95.2		16.1	4.70 × 10^3^	0.788	3.70 × 10^3^	0.0
		O6′–H (HA^2−^)				HA^2−^	1.8	2.38 × 10^9^	0.212	5.05 × 10^8^	94.8
	*k*_total_			**1.68 × 10** ^**4**^						**5.32 × 10** ^**8**^	
5CQA	SET					H_2_A^−^	38.2	15.1	0.869	5.56 × 10^−16^	0.0
						HA^2−^	6.8	6.60 × 10^7^	0.131	8.65 × 10^6^	6.8
	FHT	O6′–H	15.3	9.50 × 10^3^	49.2	H_2_A^−^	18.0	2.40 × 10^2^	0.869	2.09 × 10^2^	0.0
		O7′–H	15.0	9.80 × 10^3^	50.8		17.5	3.10 × 10^2^	0.869	2.69 × 10^2^	0.0
		O6′–H (HA^2−^)				HA^2−^	5.4	9.00 × 10^8^	0.131	1.18 × 10^8^	93.2
	*k*_total_			**1.93 × 10** ^**4**^						**1.27 × 10** ^**8**^	

Lipid environment:

2 *k*_total_ = *k*_app_(FHT(O6′–H)-neutral) + *k*_app_(FHT(O7′–H)-neutral)

Water at physiological pH:

3 *k*_total_ = *k*_f_(SET-HA^−^) + *k*_f_(FHT(O6′–H)-HA^−^) + *k*_f_(FHT(O7′–H)-HA^−^) + *k*_f_(SET-A^2−^) + *k*_f_(FHT(O6′–H)-A^2−^)

4 *k*_f_ = *k*_app_·*f*

As shown in Table 2, the calculations suggest that CQAs can be potent HOO˙ scavengers in the nonpolar environment, with *k*_total_ = 1.09 × 10^4^–1.93 × 10^4^ M^−1^ s^−1^. The rate constant for the reaction between 5CQA and HOO˙ was found to be the highest, whereas the reaction between 3CQA and HOO˙ exhibited the lowest rate constant. Based on the calculated data, the HOO˙ radical trapping ability of CQAs in the lipid medium can be ranked as follows: 5CQA > 4CQA > 3CQA. Thus, the activity of CQAs in the nonpolar medium is comparable to reference antioxidants including ascorbic acid (*k* = 5.71 × 10^3^ M^−1^ s^−1^),^40^ resveratrol (*k* = 1.31 × 10^4^ M^−1^ s^−1^),^64^ and Trolox (*k* = 3.40 × 10^3^ M^−1^ s^−1^).^68^

In water at pH = 7.40, the FHT mechanism of the neighboring hydroxyl group (O6′–H) of the dianion states determined the HOO˙ radical scavenging activity of the CQAs (*Γ* = 93.2–95.2%), while the SET reaction of these species contributed approximately 4.8–6.8% to the *k*_total_. It should be noted that the tunneling corrections (*κ*) had a negligible impact on the H-abstraction rate constant of the dianion state, the substantial imaginary frequencies (*ν* > 3000 cm^−1^) of these transition states notwithstanding. This suggests that the remarkably swift reaction rates are caused solely by the excessively low Gibb activation energy (Δ*G*^≠^ = 1.0–5.4 kcal mol^−1^) (*k*_TST_ ≈ *k*_D_, where *k*_D_ denotes the diffusion rate). The HOO˙ radical scavenging activity of the CQAs was not influenced by the monoanion states, despite the fact these states exist about 13.1% to 21.2% (Table 1) of the CQAs in water at pH = 7.4. All compounds exhibit outstanding HOO˙ antiradical activity with *k*_total_ ≈ 10^8^ M^−1^ s^−1^. 4CQA had the maximum activity with *k*_total_ = 5.32 × 10^8^ M^−1^ s^−1^ that is approximately 4.19 and 1.05 times faster than 5CQA and 3CQA, respectively. In water at pH = 7.4, the radical trapping activity of CQAs against HOO˙ is ranked as follows: 4CQA > 5CQA > 3CQA. In water at physiological pH, the CQAs demonstrated ≈ 10^4^ times greater HOO˙ radical scavenging ability than in the nonpolar environment. In water, CQAs have greater HOO˙ antiradical capacity than Trolox (*k* = 8.96 × 10^4^ M^−1^ s^−1^),^68^ resveratrol (*k* = 5.62 × 10^7^ M^−1^ s^−1^),^64^ and ascorbic acid (*k* = 9.97 × 10^7^ M^−1^ s^−1^),^40^ but the fairly similar activity to caffeic acid (*k* = 2.69 × 10^8^ M^−1^ s^−1^), ferulic acid (*k* = 3.36 × 10^8^ M^−1^ s^−1^) and dihydrocaffeic acid (*k* = 1.04 × 10^8^ M^−1^ s^−1^).^63^ Consequently, CQAs are promising natural antioxidants.

**Fig. 5:**
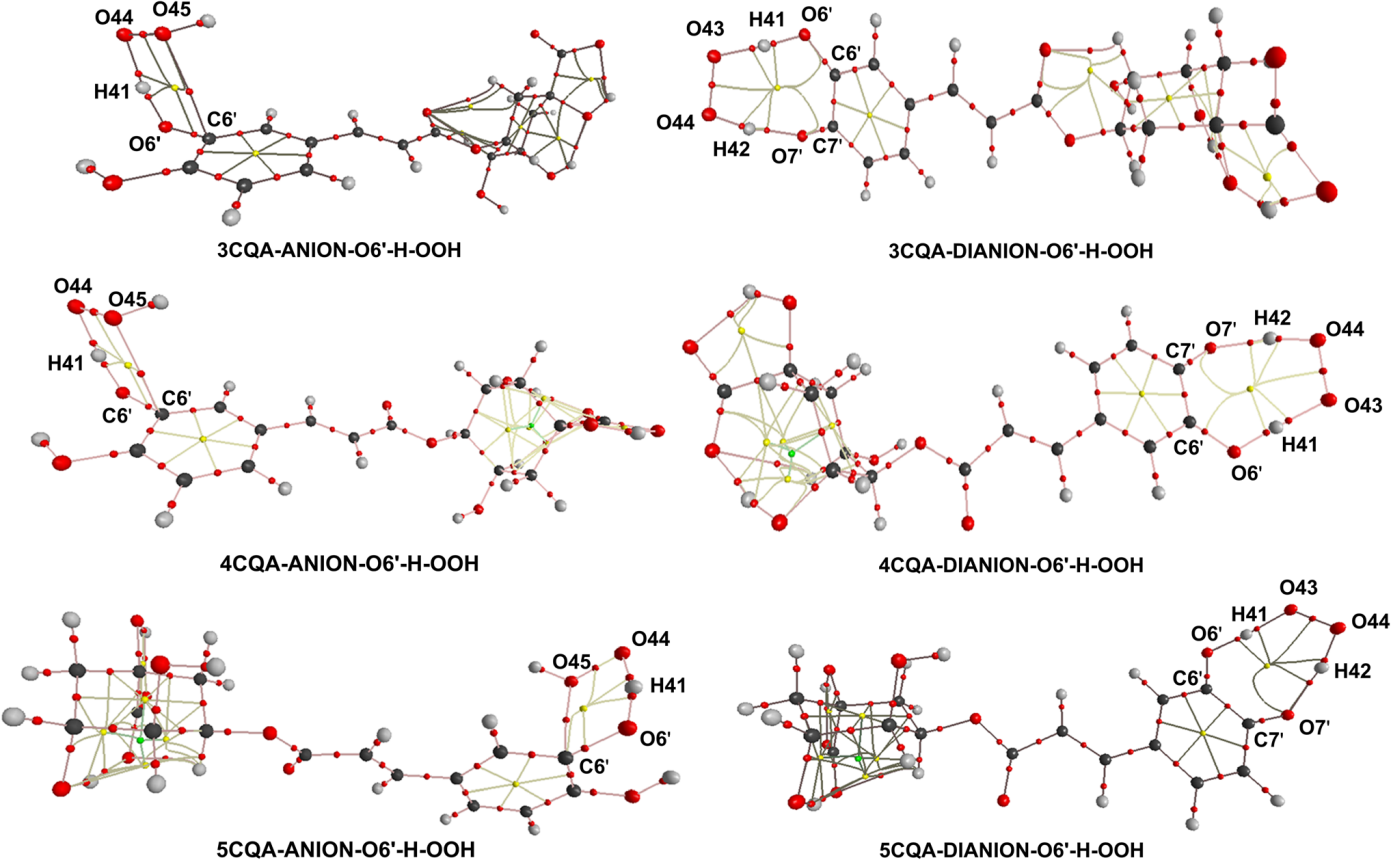
AIM topological structures of the FHT TSs of the O6′–H bond of the anionic and dianionic states. The bond critical points (BCPs) are represented by red spheres, while the ring critical points (RCPs) are represented by yellow spheres.

According to the information provided above, the removal of a hydrogen atom from the neighboring hydroxyl group (O6′–H bond) of the dianion states is responsible for the scavenging activity of CQAs in water at a pH level that is characteristic of physiological conditions. In this section, AIM analysis was employed to examine the structural characteristics of the transition states (TSs) pertaining to the O6′–H bond. The findings are displayed in Table S3, ESI,† and Fig. 5. The analysis of the AIM data reveals that the stability of the TSs-DIANION-O6′-H-OOH species can be attributed to intermolecular interactions occurring at certain sites, namely O6′⋯H41, O43⋯H41, O7′⋯H42, and PCR(O6′–H41–O43–O44–H42–O7′–C7′–C6′) (Fig. 5), whereas the stability of TSs-ANION-O6′–H–OOH is given by the intramolecular hydrogen bonding at H41⋯O6′, H41⋯O44 and O45⋯C6′ and at PCR(C6′–O6′–H41–O44–O45). The formation of an intramolecular hydrogen bond between O7′ and H42 in the TSs-DIANION-O6′-H-OOH results in the creation of an 8-atom ring, which includes O6′–H41–O43–O44–H42–O7′–C7′–C6′ (Fig. 5). The electron density between O7′ and H42 exhibits characteristics of partial covalent bonding, as evidenced by ∇^2^*ρ*(*r*) > 0, *G*(*r*)/|*V*(*r*)| ≤ 1 and *H*(*r*) < 0.^69,70^ This electron density significantly contributes to the stability of the transition state, as revealed by the substantial negative values of *E*_HD_ (−26.9 to −27.9 kcal mol^−1^). Thus, the total E_HB_ of TSs-DIANION-O6′–H–OOH (*E*_HB_ = −211.0 to −212.9 kcal mol^−1^) is about 1.13 times lower than those of TSs-ANION-O6′–H–OOH (*E*_HB_ = −184.6 to −186.7 kcal mol^−1^, respectively). This underpins the increased stability of TSs-DIANION-O6′–H–OOH and consequently the decreased Δ*G*^≠^ values (Δ*G*^≠^ = 1.0–5.4 kcal mol^−1^) in comparison with the remaining TSs.

### 3.3. The effect of pH values on the reactions of CQAs with HOO˙ in water

**Fig. 6:**
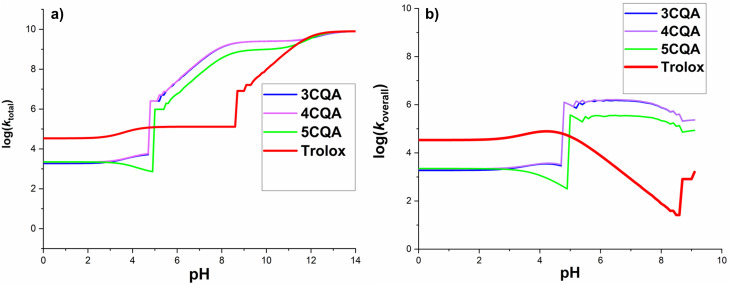
Calculated log(*k*_total_) (a) and log(*k*_overall_) (b) at 298.15 K, in the CQAs + HOO˙ in water as a function of pH values.

The impact of solution pH on the rate constants was also evaluated. Eqn (5)–(8) were employed in the computation of several key parameters, namely the rate constant (*k*), the rate constant specific to each protonation state (*k*_state_), the total rate constant (*k*_total_), and the overall rate constant (*k*_overall_). The outcomes are displayed in Fig. 6 and Table 3.

5 *k* = *k*_app_(SET) + ∑*k*_app_(FHT)

6 *k*_state_ = *k*·*f*(CQA)

7 *k*_total_ = ∑*k*_state_

8 *k*_overall_ = *f*(HOO˙)·*k*_total_

**Table 3:** Calculated Δ*G*^≠^ (kcal mol^−1^), *k*_app_ and *k* (M^−1^ s^−1^) of the CQAs + HOO˙ reactions in the water

Comp.	States	Mechanisms		Δ*G*^≠^	*k*_app_	*k*
3CQA	H_3_A	FHT	O6′–H	17.4	5.80 × 10^2^	1.86 × 10^3^
			O7′–H	17.1	1.28 × 10^3^	
	H_2_A^−^	SET		34.3	4.80 × 10^−13^	5.80 × 10^3^
		FHT	O6′–H	15.9	4.80 × 10^3^	
			O7′–H	16.6	1.00 × 10^3^	
	HA^2−^	SET		6.4	1.20 × 10^8^	2.52 × 10^9^
		FHT	O6′–H	1.0	2.40 × 10^9^	
	A^3−^	SET		0.0	8.10 × 10^9^	8.10 × 10^9^
4CQA	H_3_A	FHT	O6′–H	16.3	5.00 × 10^1^	2.15 × 10^3^
			O7′–H	16.6	2.01 × 10^3^	
	H_2_A^−^	SET		32.3	1.40 × 10^−11^	6.80 × 10^3^
		FHT	O6′–H	16.2	2.10 × 10^3^	
			O7′–H	16.1	4.70 × 10^3^	
	HA^2−^	SET		6.4	1.30 × 10^8^	2.51 × 10^9^
		FHT	O6′–H	1.8	2.38 × 10^9^	
	A^3−^	SET		0.0	8.10 × 10^9^	8.10 × 10^9^
5CQA	H_3_A	FHT	O6′–H	17.0	1.80 × 10^3^	2.23 × 10^3^
			O7′–H	18.0	4.30 × 10^2^	
	H_2_A^−^	SET		38.2	6.40 × 10^−16^	5.50 × 10^2^
		FHT	O6′–H	18.0	2.40 × 10^2^	
			O7′–H	17.5	3.10 × 10^2^	
	HA^2−^	SET		6.8	6.60 × 10^7^	9.66 × 10^8^
		FHT	O6′–H	5.4	9.00 × 10^8^	
	A^3−^	SET		0.0	8.30 × 10^9^	8.30 × 10^9^

The log(*k*_total_) for the total rate constant (Fig. 6a) did not change below pH 4.7, however, it increased significantly between pH = 4.8 and 8.3 by 4–6 units and afterward grew progressively until pH 14. The sudden increase in the log(*k*_total_) figures at pH = 4.8 and 8.3 is due to the appearance of HA^2−^ states and thus the onset of rapid SET processes. In acidic media (pH < 4.7), the reactions between CQAs and HOO˙ are sluggish because the majority of the CQAs exist in the H_3_A states (neutral states), acting *via* a slow FHT reaction.

It was demonstrated that p*K*_a_(HOO˙) is 4.80, and thus the *f*(HOO˙) value is zero at pH > 9.1. Since only pH levels below 9.1 had any impact on the *k*_overall_ values of reactions between CQAs and HOO˙, only these were looked at (Fig. 6b). It was found that as the pH levels rose, the *k*_overall_ changed. Most of the studied acids showed a rise in log(*k*_overall_) at pH 4; after a brief fall, the log(*k*_overall_) significantly rose at pH = 4.7–6.5, before decreasing once again. For this range, *k*_overall_ was 0 because *f*(HOO˙) = 0 at pH > 9.2 (Fig. 6b).

In terms of the CQAs, the exhibition was fairly similar to the HOO˙ antiradical activity at pH < 3, however at the rest pH values, 5CQA reacted with the HOO˙ lower than 3CQA or 4CQA. It is important to notice that the 3CQA acid had a fairly similar HOO˙ radical scavenging activity to 4CQA in all of the studied pH levels. Compared with typical antioxidant-Trolox, at pH 4.7, CQAs had less HOO˙ radical scavenging activity than Trolox; nevertheless, at pH > 5.0, these acids reacted with the HOO˙ more quickly than the standard. According to the calculated data, in the pH range of 5.0–8.6, the CQAs had the highest HOO˙ antiradical activity (log(*k*_overall_) = 5.1–6.2). It was found that the calculated rate constant for the 5CQA + HOO˙ reaction (*k*_overall_(calculation) = 3.10 × 10^5^ M^−1^ s^−1^) exhibit a high level of consistence with the empirical observations (*k*_exp_ = 1.28 × 10^5^ M^−1^ s^−1^, pH = 7.5).^17^ Therefore, the computed kinetic values are fairly accurate.

## 4. Conclusion

DFT calculations were performed to examine the effectiveness of monocaffeoylquinic acids in scavenging hydroperoxyl radicals. In water at physiological pH, the CQAs demonstrated ≈ 10^4^ times (*k*(water, pH = 7.4) = 1.27–5.32 × 10^8^ M^−1^ s^−1^) greater HOO˙ radical-trapping activity than in the nonpolar environment (*k*(lipid) = 1.09–1.93 × 10^4^ M^−1^ s^−1^). The FHT reaction of the neighboring hydroxyl group (O6′–H) of the dianion states determined the activity in the aqueous solution (*Γ* = 93.2–95.2%), while the SET mechanism of these states contributed 4.8–6.8% to the total rate constants. It is significant that the computed rate constant of the HOO˙ radical-trapping activity in water at pH 7.5 agrees favorably with experimental findings (*k*_calculated_/*k*_experimental_ = 2.4), supporting the computational method. CQAs exhibited similar HOO˙ antiradical activities at pH < 3, however at higher pH values, 5CQA reaction with HOO˙ was slower than that of 3CQA or 4CQA. It was also found that CQAs had less HOO˙ radical scavenging activity than Trolox at pH 4.7 while at pH > 5.0 CQAs are better radical scavengers than the reference. The CQAs had the highest HOO˙ antiradical activity at pH = 5.0–8.6. Thus, in the physiological environments, the HOO˙ antiradical ability of CQAs is generally better than the reference antioxidants resveratrol, ascorbic acid, and Trolox.

## Conflicts of interest

There are no conflicts to declare.

## Supplementary Material

RA-014-D3RA08460D-s001
