# Supplementary material for: The radical scavenging activity of monocaffeoylquinic acids: the role of neighboring hydroxyl groups and pH levels
Source: RSC Adv. 2024 Jan 30;14(6):4179–87. doi: 10.1039/d3ra08460d (PMC10825902; doi:10.1039/d3ra08460d)
Supplement: RA-014-D3RA08460D-s001 [file RA-014-D3RA08460D-s001.pdf]

Electronic Supplementary Material (ESI) for RSC Advances.
This journal is © The Royal Society of Chemistry 2024

Supporting Information (SI)

The radical scavenging activity of monocaffeoylquinic acids: The role of neighboring hydroxyl groups and pH levels

Quan V. Vo,[1*] Duong Thi Thuy Hoa[2], Nguyen Thi Hoa[1], Tran Duc Manh[2] and Adam Mechler[3]

[1]*The University of Danang - University of Technology and Education, Danang 550000, Vietnam.*

[2]*The University of Danang - University of Sciences and Education, Danang 550000, Vietnam.*

[3]*Department of Chemistry and Physics, La Trobe University, Victoria 3086, Australia.*

**Corresponding authors: vvquan@ute.udn.vn*

Table of Contents

Table S1. The method to calculate rate constant following the conventional transition state theory S2

Table S2. BDEs, PAs, and IEs (kcal/mol) of the CQAs and ΔG^o (kcal/mol) of the CQAs + HOO$^\bullet$ reactions in the examined media according to the FHT, SET, and PL mechanisms. S4

Table S3. Selected BCP intermolecular contact parameters for the TSs-O6'-H-OOH S6

Figure S1. The IRC plots for the transition states of CQAs + HOO$^\bullet$ reactions. S8

Figure S2. The typical conformers of CQA and the relative free energies ΔG^o (in kcal/mol) compared with CQA conformers (3CQA, 4CQA, 5CQA)..S9

Table S4: The Cartesian coordinates and energies of transition states of the reaction between the CQAs with HOO$^\bullet$ in the studied media (P: pentyl ethanoate; W: water) S10

References S24

Table S1. The method to calculate rate constant following the conventional transition state theory

The rate constant (k) was calculated by using the conventional transition state theory (TST) (at 298.15 K, 1M standard state) according to the equation (1):[1-5]

$$k = \sigma\kappa\frac{k_BT}{h}e^{-(\Delta G^{\neq})/RT} \qquad (1)$$

Where: σ is the reaction symmetry number ,[6,7]

κ contains the tunneling corrections calculated using the Eckart barrier,[8]

k_B is the Boltzmann constant,

h is the Planck constant,

$\Delta G^{\neq}$ is the Gibbs free energy of activation.

The Marcus Theory was used to estimate the reaction barriers of SET reactions.[9-12] The free energy of reaction $\Delta G^{\neq}$ for the SET pathway was computed following the equations (2,3).

$$\Delta G^{\neq}_{SET} = \frac{\lambda}{4}\left(1+\frac{\Delta G^{0}_{SET}}{\lambda}\right)^2 \qquad (2)$$

$$\lambda \approx \Delta E_{SET} - \Delta G^{0}_{SET} \qquad (3)$$

where ΔG_{SET} is the Gibbs energy of reaction, ΔE_{SET} is the non-adiabatic energy difference between reactants and vertical products for SET.[13,14]

For rate constants that were close to the diffusion limit a correction was applied to yield realistic results[15]. The apparent rate constants (k_{app}) were calculated following the Collins–Kimball theory in the solvents at 298.15K;[16] the steady-state Smoluchowski rate constant (k_D) for an irreversible bimolecular diffusion–controlled reaction was calculated following the literature as corroding to equations (4,5).[15,17]

$$k_{app} = \frac{k_{TST}k_D}{k_{TST}+k_D} \qquad (4)$$

$$k_D = 4\pi R_{AB}D_{AB}N_A \qquad (5)$$

where R_{AB} is the reaction distance, N_A is the Avogadro constant, and $D_{AB} = D_A + D_B$ (D_{AB} is the mutual diffusion coefficient of the reactants A and B),[16,18] where D_A or D_B is estimated using the

Stokes–Einstein formulation (6).[19,20]

$$D_{A\ or\ B} = \frac{k_{\mathrm{B}}T}{6\pi\eta a_{A\ or\ B}} \qquad (6)$$

η is the viscosity of the solvents (i.e. η(H_2O) = 8.91×10^{-4} Pa s, η(pentyl ethanoate) = 8.62×10^{-4} Pa s) and *a* is the radius of the solute.

The kinetic study requires different considerations. Water (dielectric constants, ε = 78.35) and pentyl ethanoate (ε = 4.73) are the *de facto* standard solvents in the literature to mimic the polar and nonpolar environments in the human body.[15,21-23] Thus, these solvents were used to model the physiological environments. All transition states were characterized by the existence of only one single imaginary frequency. Intrinsic coordinate calculations (IRCs) were performed to ensure that each transition state is connected correctly with the pre-complex and post-complex.

Table S2. BDEs, PAs, and IEs (kcal/mol) of the CQAs and ΔG° (kcal/mol) of the CQAs + $HOO^{\bullet}$ reactions in the examined media according to the FHT, SET, and PL mechanisms.

Comp.	positions	Pentyl ethanoate						water					
		**FHT**		**PL**		**SET**		**FHT**		**PL**		**SET**	
		**BDE**	**ΔG°**	**PA**	**ΔG°**	**IE**	**ΔG°**	**BDE**	**ΔG°**	**PA**	**ΔG°**	**IE**	**ΔG°**
	C2-RAD	99.8	13.3					100.6	10.8				
	C3-RAD	95.9	8.9					97.5	7.3				
	C4-RAD	94.4	7.7					96.5	6.5				
	C5-RAD	96.8	10.2					98.3	8.4				
	C6-RAD	101.4	14.8					102.6	12.7				
**3CQA**	COO-RAD	113.8	27.5	66.7	85.2	136.9	74.5	111.7	22.1	30.9	38.5	103.3	22.8
	O1-RAD	108.3	22.2	89.0	107.2			127.8	38.4	50.3	57.6		
	O4-RAD	106.4	20.3	104.1	122.9			108.5	19.2	58.8	66.6		
	O5-RAD	106.6	20.4	104.8	122.2			107.5	18.0	59.1	65.7		
	O61-RAD	81.1	-4.4	77.3	96.4			84.5	-4.2	42.9	51.0		
	O71-RAD	79.1	-6.2	73.7	92.9			82.8	-5.8	40.8	49.1		
	C2-RAD	97.3	11.1					97.7	8.2				
	C3-RAD	92.1	4.9					93.8	3.4				
	C4-RAD	91.3	3.3					92.8	1.5				
	C5-RAD	88.3	1.7					90.2	0.4				
	C6-RAD	97.3	11.1					97.7	8.2				
**4CQA**	COO-RAD	114.1	27.3	55.7	74.2	130.3	68.2	112.3	22.3	27.2	34.7	93.5	13.2
	O1-RAD	106.1	20.2	89.8	107.6			130.4	41.2	51.3	58.1		
	O3-RAD	107.5	20.4	88.7	108.6			108.8	18.5	49.3	58.3		
	O5-RAD	105.4	19.0	97.7	116.7			108.0	18.3	55.2	63.3		
	O61-RAD	81.3	-4.3	76.5	95.2			84.5	-4.4	43.2	51.0		
	O71-RAD	79.4	-5.9	72.6	91.5			82.7	-5.9	41.2	49.3		
	C2-RAD	99.3	12.8					100.3	10.5				
	C3-RAD	93.3	7.5					95.7	6.6				
	C4-RAD	95.9	10.4					98.0	9.1				
	C5-RAD	98.5	11.7					99.5	9.4				
	C6-RAD	99.5	12.7			139.6	76.9	100.7	10.7			102.1	21.3
**5CQA**	COO-RAD	113.9	27.9	64.0	82.7			114.4	25.2	29.0	36.8		
	O1-RAD	107.0	20.6	97.8	115.2			131.6	42.0	54.4	60.9		
	O3-RAD	107.3	21.0	92.0	111.9			108.9	19.4	51.7	60.7		
	O4-RAD	104.9	19.1	104.5	122.7			107.9	18.9	58.6	65.8		
	O61-RAD	81.9	-3.9	75.4	94.0			85.4	-3.6	42.7	50.4		

	O71-RAD	79.6	-6.1	74.0	92.7			83.2	-5.8	40.6	48.4		

Table S3. Selected BCP intermolecular contact parameters for the TSs-O6'-H-OOH

Contacts	ρ(r) (au)	∇^2ρ(r) (au)	G(r)[a] (au)	V(r)[b] (au)	G(r)/|V(r)|	H(r)[c] (au)	E_{HB}[d] (kcal/.mol)
**3CQA-ANION-O6'-H-OOH**							
O6'···H41	0.2322	0.9017	0.0894	–0.4135	0.2163	–0.3241	–129.7
O44···H41	0.1262	0.0485	0.0832	–0.1543	0.5393	–0.0711	–48.4
C6'···O45	0.0182	0.0584	0.0135	–0.0123	1.0908	0.0011	–3.9
RCP	0.0156	0.0765	0.0171	–0.0151	1.1341	0.0020	–4.7
**3CQA-DIANION-O6'-H-OOH**							
O6'···H41	0.2008	–0.6287	0.1027	–0.3626	0.2833	–0.2599	–113.8
O43···H41	0.1551	–0.1450	0.0914	–0.2191	0.4173	–0.1277	–68.7
O7'···H42	0.0751	0.1730	0.0653	–0.0874	0.7473	–0.0221	–27.4
RCP	0.0105	0.0485	0.0108	–0.0096	1.1331	0.0013	–3.0
**4CQA-ANION-O6'-H-OOH**							
O6'···H41	0.2322	–0.9399	0.0893	–0.4136	0.2160	–0.3243	–129.8
O44···H41	0.1260	0.0488	0.0831	–0.1540	0.5396	–0.0709	–48.3
C6'···O45	0.0181	0.0584	0.0134	–0.0122	1.0966	0.0012	–3.8
RCP	0.0155	0.0760	0.0170	–0.0150	1.1345	0.0020	–4.7

4CQA-DIANION-O6'-H-OOH							
O6'···H41	0.1990	−0.6012	0.1033	−0.3568	0.2894	−0.2536	−112.0
O43···H41	0.1573	−0.1615	0.0918	−0.2239	0.4098	−0.1322	−70.3
O7'···H42	0.0740	0.1729	0.0645	−0.0858	0.7518	−0.0213	−26.9
RCP	0.0105	0.0484	0.0108	−0.0096	1.1332	0.0013	−3.0
**5CQA-ANION-O6'-H-OOH**							
O6'···H41	0.2285	−0.8867	0.0900	−0.4017	0.2241	−0.3117	−126.0
O44···H41	0.1293	0.0357	0.0842	−0.1594	0.5280	−0.0753	−50.0
C6'···O45	0.0182	0.0590	0.0135	−0.0123	1.0981	0.0012	−3.9
RCP	0.0156	0.0765	0.0171	−0.0151	1.1343	0.0020	−4.7
**5CQA-DIANION-O6'-H-OOH**							
O6'···H41	0.1926	−0.5154	0.1041	−0.3371	0.3089	−0.2330	−105.8
O43···H41	0.1632	−0.2127	0.0922	−0.2376	0.3881	−0.1454	−74.5
O7'···H42	0.0754	0.1733	0.0656	−0.0879	0.7465	−0.0223	−27.6
RCP	0.0107	0.0490	0.0110	−0.0097	1.1300	0.0013	−3.1

a) electron kinetic energy density; b) electron potential energy density; c) total electron energy density; d) individual energies of each hydrogen bond.

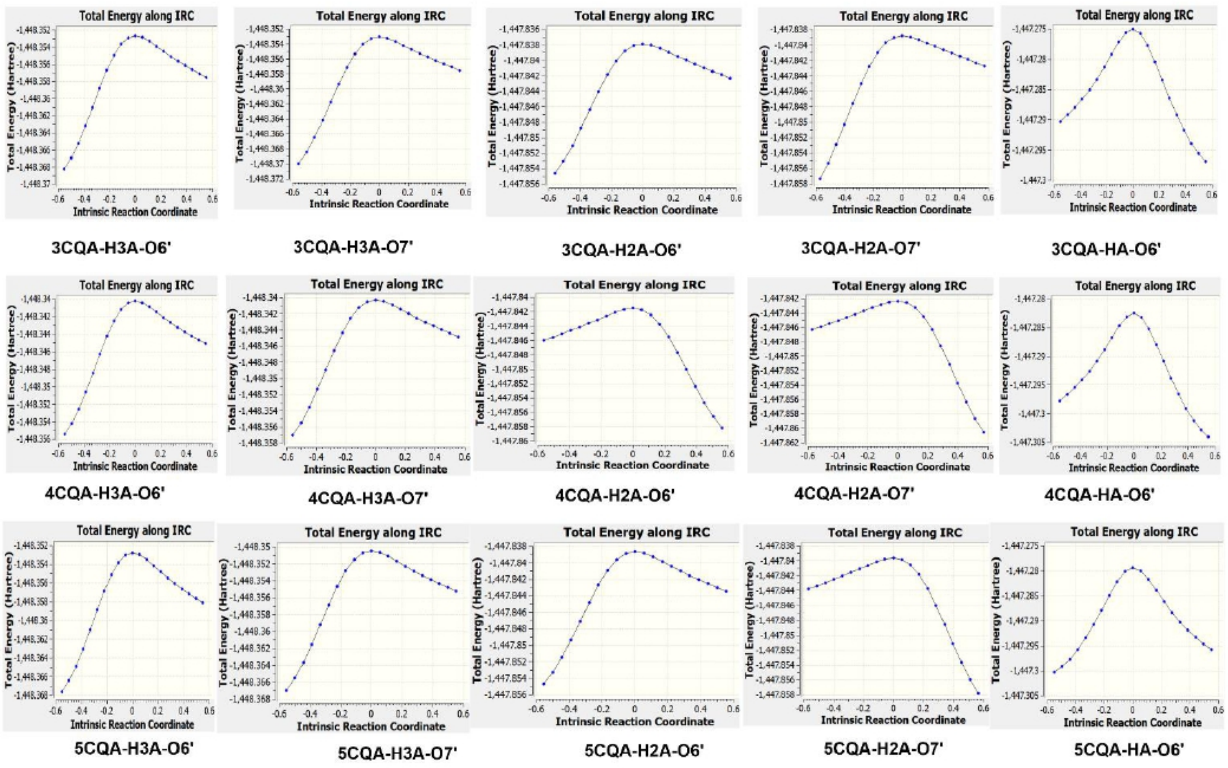

Figure S1. The IRC plots for the transition states of CQAs + HOO$^{\bullet}$ reactions.

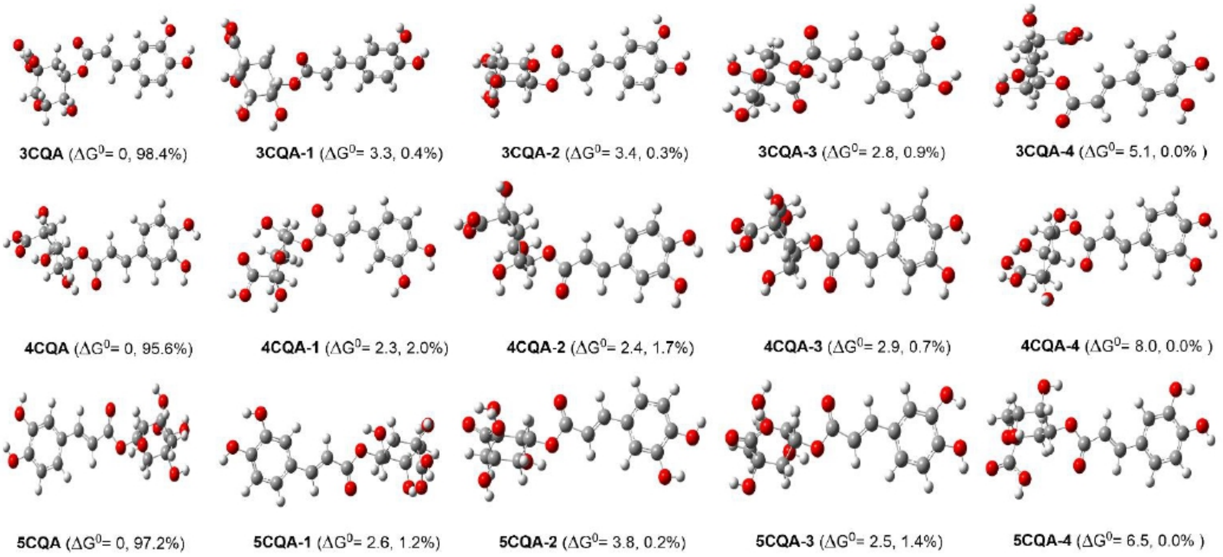

Figure S2. The typical conformers of CQA and the relative free energies $\Delta G°$ (in kcal/mol) compared with CQA conformers (3CQA, 4CQA, 5CQA)

Table S4: The Cartesian coordinates and energies of transition states of the reaction between the CQAs with HOO• in the studied media (P: pentyl ethanoate; W: water)

**Name**				**3CQA-O6’-H-OOH-P**
Cartesian Coordinates				Frequency and Energy
O	1.39807900	-0.84195200	0.73943600	Zero-point correction= 0.351552 (Hartree/Particle)
O	5.43114500	0.16832800	1.36928400	Thermal correction to Energy= 0.377817
O	2.77676800	-2.85098600	-0.74976100	Thermal correction to Enthalpy= 0.378762
O	5.35064000	-2.26039200	0.01674100	Thermal correction to Gibbs Free Energy= 0.291733
O	5.44937700	2.77953900	1.31510400	Sum of electronic and zero-point Energies= -1448.040192
O	5.23836500	2.85049800	-0.91138900	Sum of electronic and thermal Energies= -1448.013926
O	0.74357400	0.15291900	-1.17532700	Sum of electronic and thermal Enthalpies= -1448.012982
O	-6.64098400	0.27123700	-1.41572800	Sum of electronic and thermal Free Energies= -1448.100010
O	-7.37053400	-0.95431800	0.86646600	
C	4.79568500	0.76611800	0.24381400	
C	2.80013000	-0.73343200	0.43850600	
C	3.26326800	0.72123300	0.38498800	
C	5.24614600	-0.01421300	-0.99020100	
C	3.20734200	-1.51012200	-0.81624300	
C	4.73295100	-1.45102300	-0.98094300	
C	5.19876800	2.23471600	0.11677500	
C	0.48131700	-0.39909000	-0.13353500	
C	-0.88292000	-0.67212300	0.36969600	
C	-1.94053800	-0.27827000	-0.34770200	
C	-3.35126700	-0.46369700	-0.00679200	
C	-4.31575600	0.00454300	-0.88738800	
C	-3.77194600	-1.10659200	1.18359800	
C	-5.68256600	-0.15008100	-0.60530900	
C	-5.10733400	-1.27645200	1.48181500	
C	-6.07547800	-0.80002000	0.59530300	
H	3.25873300	-1.22213100	1.30164100	
H	2.81349800	1.23268200	-0.46821300	
H	2.95739300	1.23860000	1.29993800	
H	6.33749700	-0.00956900	-1.04701900	
H	4.86031700	0.49334700	-1.87641300	
H	2.73998600	-1.06384300	-1.69484100	
H	4.97620700	-1.92212700	-1.93584100	
H	5.25670800	0.69407800	2.15978000	
H	3.40274500	-3.31233600	-0.17582200	
H	5.53037000	-1.70554900	0.78680400	
H	5.65289800	3.72053500	1.19510600	
H	-0.95961900	-1.19377000	1.31547700	
H	-1.74663300	0.23206700	-1.28842900	
H	-4.03410700	0.49525600	-1.81371700	
H	-3.03247600	-1.47839600	1.88271000	
H	-5.42337200	-1.76950300	2.39293200	
H	-6.78461300	1.35069000	-1.25009100	
H	-7.88003800	-0.58774800	0.12586200	
H	-5.20767800	2.47652100	0.31606700	
O	-6.76323300	2.52830600	-0.71497200	

O -6.15127500 2.29460000 0.47211600	
**Name**	**3CQA-O7’-H-OOH-P**
Cartesian Coordinates	Frequency and Energy
O -1.38916300 0.54916200 0.78192500	Zero-point correction= 0.351542
O -5.52375200 0.05656000 1.36899100	(Hartree/Particle)
O -2.48269300 2.84200300 -0.52279200	Thermal correction to Energy= 0.377821
O -5.11494600 2.54732800 0.20737400	Thermal correction to Enthalpy= 0.378765
O -5.90354400 -2.51302700 1.11916600	Thermal correction to Gibbs Free Energy= 0.291849
O -5.69515200 -2.44713800 -1.10800700	Sum of electronic and zero-point Energies= -1448.040407
O -0.87357700 -0.37008200 -1.21215700	Sum of electronic and thermal Energies= -1448.014128
O 6.33222500 -1.80180800 -1.38626300	Sum of electronic and thermal Enthalpies= -1448.013184
O 7.27826500 -0.70088600 0.88584700	Sum of electronic and thermal Free Energies= -
C -4.97469400 -0.53523900 0.19609900	1448.100099
C -2.79372200 0.65753500 0.48978000	
C -3.45078600 -0.71156200 0.32667700	
C -5.31152000 0.39191700 -0.97080800	
C -3.08938400 1.58086500 -0.69475800	
C -4.60839500 1.74107600 -0.85354100	
C -5.57538600 -1.92088900 -0.03731100	
C -0.54303900 0.05290600 -0.13094700	
C 0.84548600 0.08794100 0.38405900	
C 1.83978500 -0.40804600 -0.36031300	
C 3.25586900 -0.45364500 0.00542000	
C 4.13455300 -1.10350000 -0.86115800	
C 3.74827000 0.13200500 1.19624200	
C 5.48809700 -1.17756400 -0.55803600	
C 5.08584500 0.06224700 1.50498700	
C 5.98332700 -0.59130100 0.63587900	
H -3.17990800 1.13257700 1.39473900	
H -3.07404700 -1.21108300 -0.56796900	
H -3.21980100 -1.33592300 1.19560500	
H -6.39326500 0.53973200 -1.01937100	
H -4.99515400 -0.09296000 -1.89640900	
H -2.68297900 1.14842700 -1.60978500	
H -4.78171700 2.31309200 -1.76768100	
H -5.42712500 -0.54779800 2.11543700	
H -3.04746100 3.33725000 0.08540900	
H -5.37028000 1.96415300 0.93403700	
H -6.23348100 -3.40596500 0.93167400	
H 0.99088500 0.51882100 1.36672200	
H 1.58377700 -0.82816000 -1.32995300	
H 3.77378800 -1.55287700 -1.77934800	
H 3.07122300 0.64005500 1.87117100	
H 5.48086300 0.50015100 2.41508200	
H 7.21821200 -1.76999000 -0.99293900	
H 7.76207400 0.24566200 0.62767000	
H 6.26456500 2.10258100 -0.11601200	
O 7.98893200 1.41982100 0.09930600	
O 6.89015400 1.60229000 -0.66985600	

Name	3CQA-HA-O6'-H-OOH-W
Cartesian Coordinates	Frequency and Energy
O 1.43993400 -0.79221900 0.72140200<br>O 5.41576200 0.16818200 1.42475000<br>O 2.80768500 -2.79129600 -0.77589300<br>O 5.41370300 -2.20385100 -0.00092300<br>O 6.19806200 2.58813400 1.08109600<br>O 4.91258200 2.98470000 -0.71061700<br>O 0.77567100 0.17799400 -1.20372100<br>O -6.61166000 -0.03204300 -1.48596600<br>O -7.31310100 -0.79375700 1.03827700<br>C 4.85016800 0.79950800 0.27200000<br>C 2.85165700 -0.67987000 0.42768900<br>C 3.32311600 0.76923000 0.39428800<br>C 5.29169400 0.04227200 -0.98868700<br>C 3.25896000 -1.44734000 -0.82988600<br>C 4.78365300 -1.39653000 -0.99844300<br>C 5.35892700 2.25960100 0.20466000<br>C 0.51594700 -0.34032800 -0.13714900<br>C -0.84179600 -0.56146400 0.39620100<br>C -1.90765300 -0.24407600 -0.34825500<br>C -3.31086900 -0.39813900 0.03267500<br>C -4.28868500 -0.11797900 -0.90917600<br>C -3.70787100 -0.81575000 1.32788200<br>C -5.64841900 -0.26187500 -0.59597400<br>C -5.03697500 -0.94999500 1.66482400<br>C -6.02068300 -0.67383300 0.71175300<br>H 3.29891000 -1.18334500 1.28804100<br>H 2.89026500 1.29359400 -0.45786700<br>H 3.01775400 1.27522500 1.31383000<br>H 6.38303100 0.04590000 -1.05694500<br>H 4.89246300 0.55837400 -1.86508300<br>H 2.80295400 -0.99737900 -1.71150600<br>H 5.02153200 -1.86510800 -1.95573400<br>H 6.05047100 0.81646500 1.77601400<br>H 3.35909700 -3.24964900 -0.12652100<br>H 5.58136500 -1.63993900 0.76957800<br>H -0.91148800 -0.98907400 1.38848800<br>H -1.73551900 0.16345700 -1.34159200<br>H -4.02351600 0.20811400 -1.90909500<br>H -2.95648200 -1.02869200 2.07856800<br>H -5.33907000 -1.26085400 2.65728800<br>H -7.01006500 0.97011100 -1.30815600<br>H -7.86251500 -0.58209300 0.26631600<br>H -5.55803700 2.52421400 -0.02077400<br>O -7.22045900 2.16768500 -0.80808400<br>O -6.40920200 2.15648400 0.27827100	Zero-point correction= 0.337209 (Hartree/Particle)<br>Thermal correction to Energy= 0.363276<br>Thermal correction to Enthalpy= 0.364220<br>Thermal correction to Gibbs Free Energy= 0.277766<br>Sum of electronic and zero-point Energies= -1447.622799<br>Sum of electronic and thermal Energies= -1447.596733<br>Sum of electronic and thermal Enthalpies= -1447.595789<br>Sum of electronic and thermal Free Energies= -1447.682242
**Name**	**3CQA-HA-O7'-H-OOH-W**
Cartesian Coordinates	Frequency and Energy

O	-1.39932900	0.43570800	0.79042200	Zero-point correction= 0.337795
O	-5.48966300	0.04216900	1.41436800	(Hartree/Particle)
O	-2.41888300	2.76437000	-0.48384500	Thermal correction to Energy= 0.363713
O	-5.09047800	2.52014600	0.24552800	Thermal correction to Enthalpy= 0.364657
O	-6.64482800	-2.16769300	0.81632700	Thermal correction to Gibbs Free Energy= 0.278908
O	-5.40761100	-2.58167700	-1.00501700	Sum of electronic and zero-point Energies= -1447.622267
O	-0.87442900	-0.44944400	-1.21741200	Sum of electronic and thermal Energies= -1447.596349
O	6.42191800	-1.30403600	-1.57493700	Sum of electronic and thermal Enthalpies= -1447.595405
O	7.27451700	-0.70466200	0.95195200	Sum of electronic and thermal Free Energies= -
C	-5.01472900	-0.54628800	0.19964000	1447.681154
C	-2.80902400	0.57423600	0.49671100	
C	-3.50341700	-0.77084200	0.31754300	
C	-5.31666600	0.39814300	-0.97275400	
C	-3.07573500	1.52145100	-0.67272800	
C	-4.58740500	1.73119200	-0.83517900	
C	-5.74766500	-1.89151100	-0.01981500	
C	-0.54924400	-0.06473200	-0.11387600	
C	0.82820100	-0.07666800	0.42142300	
C	1.83966600	-0.49040400	-0.35087300	
C	3.25001700	-0.54119700	0.03408600	
C	4.17797100	-0.91434100	-0.93681500	
C	3.68906700	-0.21984900	1.34150900	
C	5.53136900	-0.95985700	-0.62673400	
C	5.02572400	-0.26035100	1.65492900	
C	5.97325700	-0.63335300	0.68201600	
H	-3.17973900	1.05136600	1.40691500	
H	-3.14971600	-1.26744700	-0.58611200	
H	-3.29224000	-1.41005900	1.17867100	
H	-6.39405400	0.57431000	-1.03484600	
H	-4.99416300	-0.08061800	-1.90042600	
H	-2.68813500	1.09746800	-1.59904800	
H	-4.73872300	2.32884800	-1.73652600	
H	-6.22160300	-0.53304900	1.69794800	
H	-2.89946300	3.23369200	0.21220100	
H	-5.34579500	1.91358800	0.95733800	
H	0.95666300	0.27174300	1.43841000	
H	1.61477200	-0.81050000	-1.36519100	
H	3.85780000	-1.16295600	-1.94228100	
H	2.97169300	0.06067700	2.10201300	
H	5.38298700	-0.01638200	2.64838600	
H	7.31330500	-1.31411300	-1.19184800	
H	7.75972700	0.17684100	0.54485800	
H	6.29709300	2.16969400	0.19576600	
O	7.96704200	1.35705100	-0.03359000	
O	6.73094100	1.64668400	-0.50249500	
**Name**				**3CQA-A-O6’-H-OOH-W**
Cartesian Coordinates				Frequency and Energy
O	1.52922800	-0.63425700	0.73724800	Zero-point correction= 0.325042
O	5.55344200	0.11545200	1.39062300	(Hartree/Particle)

O	2.83854500	-2.74912600	-0.69665500	Thermal correction to Energy= 0.347784
O	5.41875200	-2.22482400	0.02356800	Thermal correction to Enthalpy= 0.348728
O	6.42527400	2.43539500	1.15516400	Thermal correction to Gibbs Free Energy= 0.270799
O	5.33272900	2.92803300	-0.76248200	Sum of electronic and zero-point Energies= -1447.192147
O	0.87479800	0.26699400	-1.22758200	Sum of electronic and thermal Energies= -1447.169405
O	-6.45996500	0.28766400	-1.54797900	Sum of electronic and thermal Enthalpies= -1447.168461
O	-7.24137600	-0.66559300	0.98246300	Sum of electronic and thermal Free Energies= -
C	5.01694300	0.77532000	0.23893400	1447.246390
C	2.93495900	-0.58579100	0.42355900	
C	3.48946600	0.83479900	0.35052700	
C	5.40241600	-0.00924400	-1.01882600	
C	3.30022700	-1.41443600	-0.81179300	
C	4.82559700	-1.42039200	-0.99873000	
C	5.64739600	2.21004700	0.19198000	
C	0.60939800	-0.20957800	-0.14815600	
C	-0.74884600	-0.41270700	0.39246600	
C	-1.83009900	-0.07100400	-0.32857900	
C	-3.22955400	-0.22014900	0.03982400	
C	-4.21773200	0.15766800	-0.86593100	
C	-3.63903800	-0.75436700	1.28935300	
C	-5.59091500	0.00962600	-0.60659700	
C	-4.96781700	-0.89238800	1.59298100	
C	-6.01066500	-0.52419000	0.68300100	
H	3.37917100	-1.07678700	1.29357600	
H	3.08795000	1.37207400	-0.51033700	
H	3.21385400	1.37558700	1.25999800	
H	6.49286300	-0.06090000	-1.09603800	
H	5.02650900	0.54025100	-1.88574500	
H	2.82594100	-0.98480500	-1.69476400	
H	5.03381600	-1.93724400	-1.94117200	
H	6.13258000	0.83362700	1.74581900	
H	3.46734200	-3.18072000	-0.10461300	
H	5.64552500	-1.61805100	0.74905100	
H	-0.81070500	-0.85834800	1.37680500	
H	-1.63688700	0.36215300	-1.30843300	
H	-3.94205500	0.56206800	-1.83498900	
H	-2.89053900	-1.05416400	2.01439900	
H	-5.28688900	-1.29756800	2.54723500	
H	-7.46459000	0.69164000	-1.21256600	
H	-7.92386500	0.65084400	0.79255300	
O	-8.49887800	1.30410400	-0.86379400	
O	-8.31010400	1.54128900	0.46756600	
**Name**				**4CQA-O6’-H-OOH-P**
Cartesian Coordinates				Frequency and Energy
O	1.54059300	-0.32550800	0.66207800	Zero-point correction= 0.351106
O	4.36600000	2.10221700	-1.56592900	(Hartree/Particle)
O	4.29925000	-2.36033700	-0.59042800	Thermal correction to Energy= 0.377346
O	3.28877300	0.20688000	2.76153900	Thermal correction to Enthalpy= 0.378290
O	6.76305400	1.62001500	-0.66674900	Thermal correction to Gibbs Free Energy= 0.291246

O	6.39167000	-0.56813900	-0.51891900	Sum of electronic and zero-point Energies= -1448.039066
O	0.40746000	-2.26623400	0.54361200	Sum of electronic and thermal Energies= -1448.012826
O	-6.84822300	-0.72151500	0.57503200	Sum of electronic and thermal Enthalpies= -1448.011882
O	-6.91056800	1.96178300	0.34540700	Sum of electronic and thermal Free Energies= -
C	4.48961100	0.93544500	-0.77476000	1448.098925
C	2.79469900	-1.02990100	0.73611900	
C	3.65308300	-0.13881600	-1.48408900	
C	3.96345500	1.19246500	0.65080100	
C	3.27900800	-1.38868800	-0.67605700	
C	3.75103400	-0.08842900	1.45802300	
C	5.96589300	0.55288400	-0.65955000	
C	0.41230000	-1.06203200	0.57158000	
C	-0.77510900	-0.18556200	0.51770300	
C	-1.99681300	-0.72852200	0.46996700	
C	-3.26918600	-0.00840900	0.42295700	
C	-4.45044300	-0.73099900	0.50855700	
C	-3.34054200	1.40114600	0.30062400	
C	-5.69444100	-0.07981000	0.48177100	
C	-4.54795100	2.06566700	0.27474400	
C	-5.73538800	1.33598800	0.36580500	
H	2.65790600	-1.94558600	1.31194200	
H	4.14093900	-0.43979600	-2.41438500	
H	2.72389600	0.36939700	-1.75523700	
H	3.00449400	1.70617000	0.53327400	
H	4.63860700	1.86006100	1.19384100	
H	2.44650100	-1.87249200	-1.19238700	
H	4.69845900	-0.61768600	1.59310300	
H	4.93158100	2.79254500	-1.19939300	
H	5.15226100	-1.90992900	-0.51834100	
H	2.40612800	0.58715600	2.67815000	
H	7.68060900	1.33023800	-0.53670900	
H	-0.59583100	0.88233800	0.53118300	
H	-2.06546600	-1.81391000	0.47899900	
H	-4.43822200	-1.81166700	0.60800900	
H	-2.42750600	1.97861600	0.21971300	
H	-4.59741000	3.14319000	0.17752600	
H	-7.11519400	-1.10562600	-0.42490000	
H	-7.61050100	1.29650600	0.44571200	
H	-5.43766300	-0.81899600	-2.24916600	
O	-7.16109600	-1.28206300	-1.70128700	
O	-6.28551800	-0.35023400	-2.15261900	
**Name**				**4CQA-O7’-H-OOH-P**
Cartesian Coordinates				Frequency and Energy
O	1.57574900	-0.74592400	0.43367600	Zero-point correction= 0.351066
O	4.10917400	2.70116300	-0.33105000	(Hartree/Particle)
O	4.44264200	-1.66574100	-1.62971800	Thermal correction to Energy= 0.377411
O	3.34822200	-1.12648000	2.53978700	Thermal correction to Enthalpy= 0.378355
O	6.56828900	2.06038400	0.24735900	Thermal correction to Gibbs Free Energy= 0.290803
O	6.38495100	0.05364200	-0.69496100	Sum of electronic and zero-point Energies= -1448.039110

O	0.60751000	-2.45485200	-0.66409300	Sum of electronic and thermal Energies= -1448.012766
O	-6.68344400	-1.49911900	-0.68008400	Sum of electronic and thermal Enthalpies= -1448.011822
O	-7.06914600	0.67238800	0.86697800	Sum of electronic and thermal Free Energies= -
C	4.35713500	1.31411500	-0.19963200	1448.099373
C	2.88712400	-1.27960000	0.16510900	
C	3.58500800	0.64385000	-1.34425800	
C	3.86498800	0.80613600	1.16943200	
C	3.34464900	-0.86814100	-1.24199600	
C	3.78744200	-0.71829700	1.25900200	
C	5.86318100	1.05757700	-0.27365600	
C	0.51348600	-1.43179200	-0.03632800	
C	-0.74755000	-0.75071500	0.32914000	
C	-1.91583600	-1.26628700	-0.07028000	
C	-3.24469400	-0.71903000	0.20282900	
C	-4.34456100	-1.36679300	-0.35935600	
C	-3.44068200	0.42680000	1.01056900	
C	-5.62780700	-0.88324200	-0.13895000	
C	-4.70746400	0.90788400	1.24020700	
C	-5.82629600	0.26694200	0.66872700	
H	2.84947100	-2.36770500	0.22638400	
H	4.05988800	0.87198600	-2.30155600	
H	2.60828200	1.13490600	-1.34264900	
H	2.86363800	1.22524400	1.30768200	
H	4.50346500	1.18781700	1.97126200	
H	2.53542500	-1.11516000	-1.93296000	
H	4.77925800	-1.16120300	1.13154500	
H	4.62932000	3.17786300	0.32706400	
H	5.25898300	-1.22966600	-1.34846300	
H	2.43539100	-0.83198500	2.64230700	
H	7.51107400	1.82905000	0.22622100	
H	-0.66226500	0.15855700	0.91113000	
H	-1.88915000	-2.17889200	-0.66087800	
H	-4.21126200	-2.24644800	-0.97884600	
H	-2.59212300	0.92507800	1.46174300	
H	-4.87937300	1.77689100	1.86588000	
H	-7.48403300	-1.03170500	-0.39458100	
H	-7.25517400	1.54818000	0.23478400	
H	-5.32275300	2.40973400	-1.08865200	
O	-7.15184200	2.43235800	-0.71616200	
O	-6.11045500	1.95730800	-1.43932700	
**Name**				**4CQA-HA-O6'-H-OOH-W**
Cartesian Coordinates				Frequency and Energy
O	1.59867800	-0.63393600	0.57558700	Zero-point correction= 0.337497
O	4.21240100	2.62928200	-0.52755900	(Hartree/Particle)
O	4.40172800	-1.81580500	-1.45312300	Thermal correction to Energy= 0.363317
O	3.44573400	-0.91172000	2.65479500	Thermal correction to Enthalpy= 0.364261
O	6.69862700	1.97006800	-0.43528200	Thermal correction to Gibbs Free Energy= 0.278305
O	6.33882400	-0.21814400	-0.66604200	Sum of electronic and zero-point Energies= -1447.625496
O	0.52594500	-2.39025200	-0.32795600	Sum of electronic and thermal Energies= -1447.599677

O	-6.74594100	-1.00861800	-0.24254500	Sum of electronic and thermal Enthalpies= -1447.598733
O	-6.91679000	1.35334400	1.10612700	Sum of electronic and thermal Free Energies= -1447.684689
C	4.43270800	1.23349400	-0.34476200	
C	2.87769600	-1.25045100	0.32164200	
C	3.55742600	0.50446400	-1.37517300	
C	4.01286300	0.86056300	1.09036600	
C	3.29870600	-0.98559000	-1.13080400	
C	3.85271500	-0.63911000	1.31954900	
C	5.95110400	0.96970100	-0.50909800	
C	0.49504500	-1.30024200	0.20575300	
C	-0.72015400	-0.53603000	0.53362100	
C	-1.92110300	-1.01916500	0.19257600	
C	-3.21375400	-0.38367700	0.44122200	
C	-4.35948700	-1.00163800	-0.03551900	
C	-3.33740300	0.83917000	1.14791100	
C	-5.62334400	-0.43471200	0.18416600	
C	-4.56642000	1.41939600	1.37152800	
C	-5.72056000	0.79178500	0.89531600	
H	2.79224600	-2.32432300	0.49135500	
H	3.97100900	0.63800500	-2.37828300	
H	2.58645900	1.00840700	-1.35171000	
H	3.04895800	1.34564500	1.27696800	
H	4.73561300	1.26591100	1.80368900	
H	2.47522800	-1.29426900	-1.77844400	
H	4.80989800	-1.14797600	1.19047500	
H	5.08844100	3.03889800	-0.44178400	
H	5.21152300	-1.33734000	-1.15853300	
H	2.59828400	-0.47230300	2.80242500	
H	-0.57772900	0.40982000	1.04119000	
H	-1.96089300	-1.97443300	-0.32528600	
H	-4.30316200	-1.93632000	-0.58279900	
H	-2.45076000	1.33339600	1.52647900	
H	-4.66056100	2.35375000	1.91086400	
H	-7.07751700	-0.48263400	-1.14175000	
H	-7.61181000	0.77993900	0.74395000	
H	-5.34008500	0.76127400	-2.40402200	
O	-7.15088300	0.35073100	-2.15682500	
O	-6.08850100	1.16134800	-1.92555500	
**Name**				**4CQA-HA-O7'-H-OOH-W**
Cartesian Coordinates				Frequency and Energy
O	1.61468800	-0.72053700	0.40059400	Zero-point correction= 0.337308 (Hartree/Particle)
O	4.20034300	2.72167200	-0.10145900	
O	4.51666100	-1.53035500	-1.67126700	Thermal correction to Energy= 0.363148
O	3.38590000	-1.26706500	2.48651100	Thermal correction to Enthalpy= 0.364092
O	6.69281400	2.08498000	0.01679300	Thermal correction to Gibbs Free Energy= 0.278026
O	6.38618000	-0.04070000	-0.58152000	Sum of electronic and zero-point Energies= -1447.625553
O	0.62094400	-2.35827300	-0.77481600	Sum of electronic and thermal Energies= -1447.599714
O	-6.66415100	-1.40657200	-0.57534500	Sum of electronic and thermal Enthalpies= -1447.598770
O	-6.99865500	0.78192700	1.02462100	Sum of electronic and thermal Free Energies= -

C	4.44009600	1.31731500	-0.11702200	1447.684836
C	2.91902800	-1.26428700	0.10786500	
C	3.62097500	0.73765600	-1.27964900	
C	3.96913500	0.72980200	1.22746900	
C	3.38521000	-0.77605200	-1.27100800	
C	3.83868400	-0.79001100	1.22548200	
C	5.96893800	1.10087500	-0.25210600	
C	0.54439400	-1.35950600	-0.09072700	
C	-0.70553100	-0.68909200	0.31481300	
C	-1.88242600	-1.17496800	-0.09803900	
C	-3.20063300	-0.62665200	0.21833300	
C	-4.31681200	-1.26861200	-0.31582100	
C	-3.36920700	0.51625400	1.03717600	
C	-5.59217800	-0.78712600	-0.04915200	
C	-4.62692000	0.99997700	1.30229100	
C	-5.76307300	0.35887000	0.77031000	
H	2.85528100	-2.35290400	0.11163300	
H	4.06969600	1.02611600	-2.23384100	
H	2.63925600	1.21733200	-1.22095500	
H	2.98617000	1.16389500	1.43833900	
H	4.65038900	1.03907400	2.02476500	
H	2.59186900	-0.99675300	-1.98849800	
H	4.81258200	-1.25583300	1.06365300	
H	5.06145300	3.12529400	0.09345000	
H	5.30503000	-1.08693300	-1.27852400	
H	2.51602700	-0.88227900	2.65547800	
H	-0.60466800	0.18793700	0.94175100	
H	-1.87929300	-2.05995100	-0.72945500	
H	-4.20291000	-2.14420100	-0.94480900	
H	-2.50639400	1.01614600	1.45838200	
H	-4.77908300	1.87410900	1.92433200	
H	-7.47143500	-0.97345800	-0.25595800	
H	-7.33787500	1.37603200	0.18094200	
H	-5.54475200	2.30131600	-1.28907600	
O	-7.37062300	2.01995200	-0.98004900	
O	-6.20152700	1.62477100	-1.53567600	
**Name**				**4CQA-A-O6’-H-OOH-W**
Cartesian Coordinates				Frequency and Energy
O	1.68574300	-0.46180000	0.43491000	Zero-point correction= 0.324773
O	4.62119400	2.51578600	-0.83755900	(Hartree/Particle)
O	4.61900700	-2.00580800	-1.11980900	Thermal correction to Energy= 0.347744
O	3.27854700	-0.48191900	2.68800900	Thermal correction to Enthalpy= 0.348688
O	7.01905200	1.73977000	-0.54292500	Thermal correction to Gibbs Free Energy= 0.268946
O	6.52876500	-0.43322000	-0.27153400	Sum of electronic and zero-point Energies= -1447.196836
O	0.59501500	-2.32388800	-0.21466000	Sum of electronic and thermal Energies= -1447.173865
O	-6.63328600	-1.23081800	0.08155500	Sum of electronic and thermal Enthalpies= -1447.172921
O	-6.88326700	1.51948000	0.62092300	Sum of electronic and thermal Free Energies= -
C	4.71752900	1.15294400	-0.45568300	1447.252663
C	2.95819100	-1.14651100	0.38212800	

C	3.89006400	0.32683200	-1.45513300	
C	4.12448800	1.00816300	0.95861000	
C	3.53968800	-1.10873600	-1.03921500	
C	3.85568500	-0.43209100	1.38601400	
C	6.23795800	0.77697900	-0.43083600	
C	0.57266300	-1.16527900	0.12671300	
C	-0.63155200	-0.33120700	0.28216100	
C	-1.85118600	-0.86537500	0.08812000	
C	-3.14474100	-0.21839900	0.21716500	
C	-4.30181800	-0.97042800	0.02410500	
C	-3.28710400	1.15388900	0.55145100	
C	-5.59322200	-0.43844400	0.17089600	
C	-4.52773600	1.71973900	0.67870300	
C	-5.73905600	0.97568300	0.49652900	
H	2.80655400	-2.18563000	0.67590100	
H	4.40025600	0.30160600	-2.42229800	
H	2.95342500	0.87818900	-1.59076800	
H	3.17635900	1.55875100	0.96008200	
H	4.78886600	1.48568100	1.68414800	
H	2.76263100	-1.48444600	-1.71278800	
H	4.79488600	-0.98392500	1.44422200	
H	5.55564200	2.79556500	-0.84171800	
H	5.42075600	-1.52246600	-0.79289100	
H	2.39708700	-0.10319100	2.60365000	
H	-0.47320000	0.70100200	0.56762700	
H	-1.87167100	-1.91726700	-0.19166000	
H	-4.23057900	-2.02508000	-0.22345500	
H	-2.40262600	1.76300700	0.70210500	
H	-4.64378400	2.76842600	0.92979400	
H	-7.58455300	-0.78098200	-0.34819800	
H	-7.66773000	1.25445300	-0.63025700	
O	-8.56580000	-0.34787500	-0.98337700	
O	-8.15264900	0.86901200	-1.44351400	
**Name**				**5CQA-O6'-H-OOH-P**
Cartesian Coordinates				Frequency and Energy
O	-1.23061200	-0.56911100	-0.46198600	Zero-point correction= 0.351323
O	-5.10258600	-0.01401900	2.38278300	(Hartree/Particle)
O	-2.94414600	-2.75440900	-0.22848600	Thermal correction to Energy= 0.377508
O	-5.20762900	-0.70044100	-2.13186300	Thermal correction to Enthalpy= 0.378452
O	-4.93392800	2.38781100	1.37650900	Thermal correction to Gibbs Free Energy= 0.292101
O	-5.29001900	1.65328400	-0.69318300	Sum of electronic and zero-point Energies= -1448.039688
O	-0.88137200	1.64883400	-0.38735500	Sum of electronic and thermal Energies= -1448.013503
O	5.99317100	-1.60598000	-0.54649000	Sum of electronic and thermal Enthalpies= -1448.012558
O	7.49649700	0.62170500	-0.52708100	Sum of electronic and thermal Free Energies= -
C	-4.66395700	0.05237700	1.03952200	1448.098909
C	-2.65401300	-0.35727900	-0.45670200	
C	-3.13216300	-0.10338800	0.96802600	
C	-5.36555600	-1.10207900	0.31116400	
C	-3.27095500	-1.62885800	-1.02524900	

C	-4.79935600	-1.51789300	-1.05350400	
C	-5.01702800	1.42431200	0.46028400	
C	-0.44482600	0.52502300	-0.42612500	
C	0.98269800	0.14954700	-0.44481600	
C	1.91506100	1.10842100	-0.41422700	
C	3.36622200	0.92210300	-0.43595400	
C	3.96991000	-0.32837400	-0.47399500	
C	4.18857700	2.07269000	-0.42617200	
C	5.36655600	-0.44213300	-0.50168800	
C	5.56756400	1.99047500	-0.45776100	
C	6.17011800	0.73555000	-0.49464100	
H	-2.88012300	0.48577500	-1.11513400	
H	-2.88135700	-0.96989900	1.58455900	
H	-2.64074600	0.77845100	1.38690300	
H	-6.43348800	-0.89084600	0.21607900	
H	-5.25837200	-1.95747400	0.98256900	
H	-2.92042300	-1.76651600	-2.05357700	
H	-5.17957600	-2.51941200	-1.26773000	
H	-4.73576500	0.73308100	2.87081500	
H	-1.98496700	-2.84478000	-0.21736000	
H	-5.28068000	0.21230400	-1.82090800	
H	-5.10715700	3.24388200	0.95248000	
H	1.21240500	-0.90810000	-0.49156000	
H	1.57205800	2.13956600	-0.37464600	
H	3.39099300	-1.24535100	-0.48883300	
H	3.71627600	3.04867800	-0.39640600	
H	6.18756800	2.87832800	-0.45124700	
H	6.03246200	-2.01912100	0.48186300	
H	7.71839100	-0.32218700	-0.57580500	
H	4.83244700	-0.75373200	2.26066000	
O	6.00080500	-2.12077900	1.75856700	
O	5.79568600	-0.83759700	2.14712300	
**Name**				**5CQA-O7'-H-OOH-P**
Cartesian Coordinates				Frequency and Energy
O	-1.38172800	-0.06200300	0.39723000	Zero-point correction= 0.350826
O	-5.69234200	1.64674200	1.57713400	(Hartree/Particle)
O	-3.10281500	-1.82245200	1.78891000	Thermal correction to Energy= 0.377265
O	-4.98840800	-1.85099600	-1.27908800	Thermal correction to Enthalpy= 0.378209
O	-5.40895300	2.78623400	-0.74748200	Thermal correction to Gibbs Free Energy= 0.290530
O	-5.31851600	0.83869600	-1.81862500	Sum of electronic and zero-point Energies= -1448.039019
O	-0.76881600	-0.85759100	-1.61739700	Sum of electronic and thermal Energies= -1448.012580
O	5.55382800	0.54016400	2.27874600	Sum of electronic and thermal Enthalpies= -1448.011636
O	7.36435900	-0.43102900	0.54564800	Sum of electronic and thermal Free Energies= -
C	-5.02481700	0.86863300	0.60294100	1448.099315
C	-2.77465200	-0.16807800	0.04466300	
C	-3.50216500	0.90103200	0.84477000	
C	-5.57637800	-0.55613800	0.75427600	
C	-3.27529400	-1.56570600	0.40527600	
C	-4.77357000	-1.69339300	0.10814000	

C -5.28366800 1.45998100 -0.78430100<br>C -0.47475700 -0.44785600 -0.52347800<br>C 0.89857000 -0.29135500 0.00215300<br>C 1.93918700 -0.66304000 -0.75236600<br>C 3.35254200 -0.58130900 -0.38482600<br>C 3.77992000 -0.03450800 0.82850200<br>C 4.30178400 -1.08190200 -1.30342800<br>C 5.13176800 0.01619800 1.12238300<br>C 5.64880200 -1.04050500 -1.01831100<br>C 6.09062700 -0.49015800 0.19869400<br>H -2.87472800 -0.01304400 -1.03217700<br>H -3.35565400 0.71541900 1.91124900<br>H -3.09826100 1.88983900 0.60940200<br>H -6.61113900 -0.59443700 0.40520400<br>H -5.58611900 -0.73545300 1.83199400<br>H -2.74236400 -2.30765600 -0.19847400<br>H -5.10400900 -2.62439100 0.57453600<br>H -5.43322600 2.57048700 1.47575000<br>H -2.16628000 -1.74117100 1.99999900<br>H -5.09377300 -0.97793300 -1.68076400<br>H -5.51998300 3.11864500 -1.65294100<br>H 0.99922500 0.11999700 0.99914700<br>H 1.72776500 -1.07358700 -1.73675000<br>H 3.07810300 0.36343300 1.55140700<br>H 3.95554300 -1.50641600 -2.23862900<br>H 6.38887400 -1.43042300 -1.70854400<br>H 6.52012900 0.46209500 2.30943700<br>H 7.83511300 0.40564700 0.01013100<br>H 6.43165100 1.60923500 -1.66125800<br>O 8.00096600 1.50641000 -0.65552200<br>O 6.73013900 1.95061600 -0.79946200	
**Name**	**5CQA-HA-O6'-H-OOH-W**
Cartesian Coordinates	Frequency and Energy
O 1.30314100 0.54650600 -0.47455900<br>O 5.11047200 0.18534200 2.42918500<br>O 3.06625100 2.71778000 -0.43298100<br>O 5.26438700 0.38516800 -2.10898200<br>O 5.42269900 -2.23072000 1.60314000<br>O 5.14295700 -1.75004100 -0.55678800<br>O 0.89719400 -1.66301700 -0.42968600<br>O -5.84944400 1.80751300 -0.11739600<br>O -7.44254300 -0.31259700 -0.72098400<br>C 4.74076900 -0.00859800 1.06759900<br>C 2.72716600 0.30542300 -0.47184900<br>C 3.20805800 0.12467500 0.96107700<br>C 5.44232600 1.08802800 0.25019300<br>C 3.36431100 1.51820300 -1.13540000<br>C 4.88868100 1.36390500 -1.15238800<br>C 5.14860800 -1.44895500 0.66563600	Zero-point correction= 0.337567 (Hartree/Particle)<br>Thermal correction to Energy= 0.363318<br>Thermal correction to Enthalpy= 0.364262<br>Thermal correction to Gibbs Free Energy= 0.279115<br>Sum of electronic and zero-point Energies= -1447.624652<br>Sum of electronic and thermal Energies= -1447.598901<br>Sum of electronic and thermal Enthalpies= -1447.597957<br>Sum of electronic and thermal Free Energies= -1447.683104

C 0.48976300 -0.51953900 -0.44900700	
C -0.92489600 -0.10979700 -0.45664300	
C -1.88646700 -1.03832300 -0.52200900	
C -3.32869000 -0.79188600 -0.54738700	
C -3.88822700 0.45102200 -0.28547400	
C -4.18585800 -1.87744900 -0.84836000	
C -5.27575100 0.62942200 -0.34645500	
C -5.55663800 -1.72929700 -0.91438700	
C -6.11526000 -0.47673000 -0.66798700	
H 2.92828300 -0.57761100 -1.07951700	
H 2.92330400 1.01078200 1.53455200	
H 2.72838000 -0.74552600 1.41692700	
H 6.51195400 0.87259400 0.18032900	
H 5.33079500 2.00901600 0.82936500	
H 3.00780900 1.58875600 -2.16793400	
H 5.30340300 2.31396900 -1.49701500	
H 5.28169900 -0.70532900 2.77572000	
H 2.10947200 2.84131300 -0.43805300	
H 5.21684700 -0.48828000 -1.65792500	
H -1.12338200 0.95456200 -0.42803300	
H -1.58777500 -2.08221800 -0.57955000	
H -3.28306500 1.31219600 -0.02612300	
H -3.74545100 -2.85040000 -1.03672400	
H -6.20653100 -2.56317200 -1.14852900	
H -6.25101700 1.79942600 0.90505000	
H -7.65754300 0.61825300 -0.54641900	
H -5.28691200 0.12621600 2.46668900	
O -6.58215100 1.43296100 2.11397100	
O -6.19845700 0.13159900 2.12308600	
**Name**	**5CQA-HA-O7’-H-OOH-W**
Cartesian Coordinates	Frequency and Energy
O -1.35552500 -0.04064500 0.36328900	Zero-point correction= 0.337124
O -5.52350400 2.07139100 1.24055200	(Hartree/Particle)
O -3.10675500 -1.42540700 2.11496800	Thermal correction to Energy= 0.362977
O -5.05190800 -1.91271100 -0.89475200	Thermal correction to Enthalpy= 0.363921
O -5.61004700 2.59652800 -1.28306800	Thermal correction to Gibbs Free Energy= 0.278350
O -5.18146400 0.50618800 -1.93021100	Sum of electronic and zero-point Energies= -1447.625062
O -0.79967800 -1.43487000 -1.31189300	Sum of electronic and thermal Energies= -1447.599209
O 5.57477700 0.91729000 1.99408800	Sum of electronic and thermal Enthalpies= -1447.598265
O 7.39460900 -0.26598800 0.34648600	Sum of electronic and thermal Free Energies= -
C -4.96437400 1.05398800 0.41528400	1447.683836
C -2.76144400 -0.17540500 0.05416800	
C -3.43421600 1.07191600 0.59957700	
C -5.55487800 -0.28268300 0.88894500	
C -3.30709700 -1.44347500 0.70741400	
C -4.81492100 -1.55388800 0.45747900	
C -5.29192400 1.40766300 -1.05810600	
C -0.47526700 -0.70884600 -0.39570700	
C 0.90865400 -0.42529900 0.02968900	

C 1.93572600 -0.98359300 -0.62211000<br>C 3.35446800 -0.77840800 -0.33104300<br>C 3.79306200 -0.00784900 0.74818000<br>C 4.29712800 -1.38347300 -1.19303100<br>C 5.15029400 0.16440400 0.96155800<br>C 5.64782300 -1.20985200 -0.99324200<br>C 6.10165500 -0.43877500 0.09017700<br>H -2.87316300 -0.24967500 -1.02850500<br>H -3.24118800 1.13608300 1.67356200<br>H -3.01369000 1.96177200 0.12411200<br>H -6.60313500 -0.35490600 0.58688100<br>H -5.53034400 -0.24916800 1.98185700<br>H -2.81851500 -2.31966900 0.27056000<br>H -5.18170100 -2.38020100 1.07098500<br>H -5.68146500 2.82286400 0.64690600<br>H -2.15847200 -1.37141000 2.28547000<br>H -5.08496200 -1.07393900 -1.40994000<br>H 1.02896500 0.25237400 0.86577900<br>H 1.71760500 -1.64382000 -1.45766000<br>H 3.09912100 0.46602600 1.43186900<br>H 3.94079200 -1.98272400 -2.02272900<br>H 6.38386300 -1.66138900 -1.64764100<br>H 6.54455400 0.90606800 2.02396000<br>H 7.68279800 0.73170400 0.01435600<br>H 6.28923200 1.71322900 -1.78165300<br>O 7.65028500 1.94059400 -0.51591100<br>O 6.34754000 2.04771100 -0.86802900	
**Name**	**5CQA-A-O6'-H-OOH-W**
Cartesian Coordinates	Frequency and Energy
O -1.39591900 0.18711200 -0.04558900<br>O -5.56462300 2.34991000 -0.68947800<br>O -2.70846200 0.84016100 2.29855700<br>O -5.11879700 -1.67905300 1.34890500<br>O -6.33169100 0.57488400 -2.32657000<br>O -5.47459100 -1.16825200 -1.20046300<br>O -0.87685800 -2.00671900 -0.03294800<br>O 5.68317000 1.72080900 -0.63674100<br>O 7.40354200 -0.49143700 -0.54946200<br>C -5.06139900 1.06054600 -0.37801100<br>C -2.81577600 -0.10028700 0.06008700<br>C -3.53075700 1.09604500 -0.54847800<br>C -5.41863600 0.75687100 1.08601700<br>C -3.15578200 -0.28011700 1.53881000<br>C -4.67006100 -0.40840500 1.74772500<br>C -5.69074300 0.05086200 -1.39607400<br>C -0.52998700 -0.85130500 -0.08083000<br>C 0.86038200 -0.36874300 -0.17916200<br>C 1.87354100 -1.25267300 -0.19368000<br>C 3.30092800 -0.99427200 -0.28154200	Zero-point correction= 0.324212 (Hartree/Particle)<br>Thermal correction to Energy= 0.346428<br>Thermal correction to Enthalpy= 0.347372<br>Thermal correction to Gibbs Free Energy= 0.270962<br>Sum of electronic and zero-point Energies= -1447.196189<br>Sum of electronic and thermal Energies= -1447.173974<br>Sum of electronic and thermal Enthalpies= -1447.173030<br>Sum of electronic and thermal Free Energies= -1447.249440

C	3.85167400	0.28405800	-0.35885300	
C	4.18278100	-2.10471700	-0.29674900	
C	5.23275200	0.50490900	-0.46914000	
C	5.54172400	-1.93657700	-0.38256900	
C	6.14226700	-0.64348400	-0.46753800	
H	-3.03775600	-1.02257000	-0.47716400	
H	-3.17951000	2.00603400	-0.05240300	
H	-3.28212500	1.17651300	-1.61008900	
H	-6.49543600	0.58260400	1.17030700	
H	-5.18073300	1.66693000	1.64558500	
H	-2.68073300	-1.19820500	1.90172600	
H	-4.83319300	-0.33932400	2.82922800	
H	-6.06014600	2.18120400	-1.51342000	
H	-1.83397200	1.07233400	1.96989400	
H	-5.25812700	-1.63934300	0.36970900	
H	1.00623600	0.70255200	-0.23693100	
H	1.58528000	-2.30096300	-0.13278800	
H	3.21961800	1.16538000	-0.36075400	
H	3.76071200	-3.10369500	-0.23806400	
H	6.21700300	-2.78501000	-0.39356000	
H	6.71077900	1.96888500	-0.18963500	
H	7.85003200	0.43157000	0.53592600	
O	7.72170900	2.29461900	0.43680400	
O	8.01435000	1.19781900	1.19478100	

References

1. M. G. Evans and M. Polanyi, *Trans. Faraday Soc.*, 1935, **31**, 875-894.
2. H. Eyring, *J. Chem. Phys.*, 1935, **3**, 107-115.
3. D. G. Truhlar, W. L. Hase and J. T. Hynes, *J. Phys. Chem.*, 1983, **87**, 2664-2682.
4. T. Furuncuoglu, I. Ugur, I. Degirmenci and V. Aviyente, *Macromolecules*, 2010, **43**, 1823-1835.
5. E. Vélez, J. Quijano, R. Notario, E. Pabón, J. Murillo, J. Leal, E. Zapata and G. Alarcón, *J. Phys. Org. Chem.*, 2009, **22**, 971-977.
6. E. Pollak and P. Pechukas, *J. Am. Chem. Soc.*, 1978, **100**, 2984-2991.
7. A. Fernández-Ramos, B. A. Ellingson, R. Meana-Pañeda, J. M. Marques and D. G. Truhlar, *Theor. Chem. Acc.*, 2007, **118**, 813-826.
8. C. Eckart, *Phy. Rev.*, 1930, **35**, 1303.
9. R. A. Marcus, *Annu. Rev. Phys. Chem.*, 1964, **15**, 155-196.
10. R. A. Marcus, *Rev. Mod. Phys.*, 1993, **65**, 599.
11. Y. Lu, A. Wang, P. Shi and H. Zhang, *PloS one*, 2017, **12**, e0169773.
12. Y. Lu, A. Wang, P. Shi, H. Zhang and Z. Li, *PloS one*, 2015, **10**, e0133259.
13. S. F. Nelsen, S. C. Blackstock and Y. Kim, *J. Am. Chem. Soc.*, 1987, **109**, 677-682.

14. S. F. Nelsen, M. N. Weaver, Y. Luo, J. R. Pladziewicz, L. K. Ausman, T. L. Jentzsch and J. J. O'Konek, *J. Phys. Chem. A*, 2006, **110**, 11665-11676.

15. A. Galano and J. R. Alvarez-Idaboy, *J. Comput. Chem.*, 2013, **34**, 2430-2445.

16. F. C. Collins and G. E. Kimball, *J. Colloid Sci.* , 1949, **4**, 425-437.

17. M. Von Smoluchowski, *Z. Phys. Chem*, 1917, **92**, 129-168.

18. D. G. Truhlar, *J. Chem. Educ.*, 1985, **62**, 104.

19. A. Einstein, *Ann. Phys.*, 1905, **17**, 549-560.

20. G. G. Stokes, *Mathematical and Physical Papers*, University Press, Cambridge, 1905.

21. A. Galano and J. Raúl Alvarez-Idaboy, *Int. J. Quantum Chem.*, 2019, **119**, e25665.

22. Q. V. Vo, T. V. Gon, M. V. Bay and A. Mechler, *J. Phys. Chem. B*, 2019, **123**, 10672-10679.

23. Q. V. Vo, N. M. Tam, M. Van Bay, N. M. Thong, T. Le Huyen, N. T. Hoa and A. Mechler, *RSC Adv.*, 2020, **10**, 14937-14943.
